# Developing a three stage coordinated approach to enhance efficiency and reliability of virtual power plants

**DOI:** 10.1038/s41598-024-63668-7

**Published:** 2024-06-07

**Authors:** Jeremiah Amissah, Omar Abdel-Rahim, Diaa-Eldin A. Mansour, Mohit Bajaj, Ievgen Zaitsev, Sobhy Abdelkader

**Affiliations:** 1 Electrical Power Engineering Department, Egypt-Japan University of Science and Technology, New Borg El-Arab City, 21934 Alexandria Egypt; 2 Electrical Engineering Department, Aswan University, Aswan, Egypt; 3 Electrical Power and Machines Engineering Department, Tanta University, Tanta, Egypt; 4 Department of Electrical Engineering, Graphic Era (Deemed to Be University), Dehradun, 248002 India; 5 Hourani Center for Applied Scientific Research, Al-Ahliyya Amman University, Amman, Jordan; 6 Graphic Era Hill University, Dehradun, 248002 India; 7 Department of Theoretical Electrical Engineering and Diagnostics of Electrical Equipment, Institute of Electrodynamics, National Academy of Sciences of Ukraine, Peremogy, 56, Kyiv-57, 03680 Ukraine; 8 Electrical Engineering Department, Mansoura University, Mansoura, 35516 Egypt; 9 Center for Information-Analytical and Technical Support of Nuclear Power Facilities Monitoring of the National Academy of Sciences of Ukraine, Akademika Palladina Avenue, 34-A, Kyiv, Ukraine

**Keywords:** Distributed energy resources, Energy markets, Optimization, Virtual power plant, Energy science and technology, Engineering, Mathematics and computing

## Abstract

A Virtual Power Plant (VPP) is a centralized energy system that manages, and coordinates distributed energy resources, integrating them into a unified entity. While the physical assets may be dispersed across various locations, the VPP integrates them into a virtual unified entity capable of responding to grid demands and market signals. This paper presents a tri-level hierarchical coordinated operational framework of VPP. Firstly, an Improved Pelican Optimization Algorithm (IPOA) is introduced to optimally schedule Distributed Energy Resources (DERs) within the VPP, resulting in a significant reduction in generation costs. Comparative analysis against conventional algorithms such as Genetic Algorithm (GA) and Particle Swarm Optimization (PSO) demonstrates IPOA's superior performance, achieving an average reduction of 8.5% in generation costs across various case studies. The second stage focuses on securing the optimized generation data from rising cyber threats, employing the capabilities of machine learning, preferably, a convolutional autoencoder to learn the normal patterns of the optimized data to detect deviations from the optimized generation data to prevent suboptimal decisions. The model exhibits exceptional performance in detecting manipulated data, with a False Positive Rate (FPR) of 1.92% and a Detection Accuracy (DA) of 98.06%, outperforming traditional detection techniques. Lastly, the paper delves into the dynamic nature of the day ahead market that the VPP participates in. In responding to the grid by selling its optimized generated power via the day-ahead market, the VPP employs the Prophet model, another machine learning technique to forecast the spot market price for the day-ahead to mitigate the adverse effects of price volatility. By utilizing Prophet forecasts, the VPP achieves an average revenue increase of 15.3% compared to scenarios without price prediction, emphasizing the critical role of predictive analytics in optimizing economic gains. This tri-level coordinated approach adopted addresses key challenges in the energy sector, facilitating progress towards achieving universal access to clean and affordable energy.

## Introduction

The sustainable progress of human civilization heavily relies on the consistent provision of sustainable energy, encompassing economic, social, and cultural aspects^1^. Achieving sustainable development necessitates the exploration and implementation of energy solutions that align with environmental preservation, technical acceptance, and economic feasibility^2^. Meeting this challenge demands comprehensive innovations addressing resource sustainability, the advancement of efficient technologies, and the adaptation of energy systems to diverse environmental, economic, and technical contexts, alongside the establishment of supportive social and legislative frameworks. Through such endeavors, sustainable energy systems can be fostered to enhance the well-being of humanity.

Distributed Energy Resources (DERs) offer a promising solution to these challenges by decentralizing energy production and distribution, thereby reducing reliance on traditional centralized power generation and transmission infrastructure^3^. With locally accessible Renewable Energy Resources (RERs) like solar, wind, DERs aid individuals to produce their own electricity in an ecologically friendly manner and aid in the reliability of the overall power grid. However, these distributed generations typically consist of small-scale generation units across various locations such as residential homes, commercial buildings, and industrial facilities which makes their impact on the overall reliability of the power grid relatively small. As such, Virtual Power Plants (VPPs) emerge as the optimal choice within this paradigm, serving as an integrated platform for managing and optimizing DERs^4^. VPPs enable the aggregation and coordination of diverse energy assets, allowing for efficient energy management, demand response, and grid stabilization. Through their flexibility and scalability, VPPs facilitate the transition towards sustainable energy systems, promoting resilience, reliability, and affordability in the provision of energy services.

VPPs play a pivotal role in shaping the future of energy systems by offering several benefits and addressing key challenges faced by modern power systems. Their importance stems from several factors such as integration of RERs, ensuring grid stability and resilience by actively managing energy generation, storage, and consumption in response to dynamic grid conditions and also facilitate participation in energy markets and enabling the monetization of DERs^5^. Additionally, VPPs contribute to enhanced grid efficiency and reliability by providing ancillary services such as frequency regulation, voltage control, and reactive power support. This multifaceted approach optimizes the utilization of available resources and minimizes wastage, thereby maximizing the overall efficiency of the energy system. Moreover, VPPs serve as a crucial mechanism for integrating intermittent renewable energy sources into the grid, mitigating the challenges associated with their variability and intermittency. By intelligently orchestrating the operation of DERs within the VPP framework, fluctuations in the energy production can be smoothed out, ensuring a stable and consistent power supply to consumers while reducing the need for back-up fossil-fuel based generation.

Furthermore, VPPs foster innovation and promote the adoption of emerging technologies such as energy storage systems, smart meters, and advanced control algorithms, driving continuous improvement and sustainability of energy systems^6^. Through the deployment of state-of-the-art monitoring and control mechanisms, VPPs enable real-time optimization of energy flows and grid operations, enhancing system resilience and adaptability to changing demand patterns. By leveraging data analytics and predictive modeling, VPPs enable proactive decision-making and adaptive strategies, optimizing energy production in alignment with evolving market dynamics. As such, VPPs serve as a cornerstone in the transition towards a more resilient, efficient, and sustainable energy future, embodying the principles of flexibility, decentralization, and innovation in the modern energy landscape.

Energy Management (EM) in a VPP is essential for maximizing the potential of DERs, guaranteeing their seamless integration with the grid, and managing the rapidly rising demand for electricity^7^. The coordination of energy generation, storage, and consumption is at the heart of VPP operations, which requires efficient energy management. Considering variables such as electricity market prices, the availability of renewable energy, changes in demand, and network constraints, the aim is to operate the VPP as efficiently as possible. However, the inherent volatility of DERs and the fluctuating demand make it difficult to achieve this optimum^8^. The VPP EM optimization is a challenging stochastic optimization. The variability of the DERs in the VPP portfolio makes the efficient use of the resources not a straightforward task. The use of metaheuristic optimization techniques as a potent tool for overcoming EM’s difficulties in VPPs has drawn a lot of attention. These methods consider the stochastic nature of the variables by describing them using probability distributions. Probabilistic techniques provide more reliable and adaptable decision-making by introducing uncertainty into the optimization models, which enhances the economic and operational performance of the VPP.

The optimization of DERs within a VPP is essential for maximizing efficiency, reducing generational and operational costs, and enhancing grid stability. By strategically coordinating the operations of DERs, VPPs minimize energy wastage, improve resource use, and minimize expenses associated with energy procurement. Optimization also enables VPPs to take part in demand response by dynamically adjusting energy consumption patterns to alleviate grid congestion and mitigate supply–demand imbalances. From a financial perspective, optimization allows the VPP to capitalize on revenue opportunities in energy markets.

For instance, to determine a composite energy system’s suitable real-time power distribution^9^, applied the multi-objective particle swarm optimization (PSO). The aim of the study was to enhance fuel economy through an energy management strategy based on PSO and double fuzzy logic control. However, the limitation of PSO in the energy management of this work is that PSO has a modest iterative convergence rate which affects the speed at which it finds optimal solutions. This limitation is very dire as real-time scenarios rely on fast and optimal solutions to aid in the decision-making process. Also, a parallel hybrid genetic algorithm (GA) is utilized in^10^ by Mellouk et al. to address the EM issue of a microgrid. The authors’ proposed an energy management strategy by adjusting Proportional Integral (P.I) controller gains to determine the power output required from battery while considering generations from PV and wind sources. Also, the GA exhibits premature convergence in the optimization process which calls for constant refinement. In^11^^,^ authors suggested a smart EM system for the operation and cost control of a multi-source-based microgrid. The suggested unit optimizes the cost of operation using Harris Hawk Optimization (HHO) algorithm following load demand, energy pricing, and generation ability. The goal is to govern various energy resources within the microgrid while keeping minimal operating expenses. One distinctive limitation of the HHO is the algorithm’s limited exploration capability in the optimization process. This limitation leads to a lack of diversity in the population and explore the solution space. In construction management engineering, authors in^12^ proposed an PSO algorithm to increase building material durability, efficiency, and cost reduction. The study used the algorithm to calculate the accuracy and material-specific energy of building materials. As discussed earlier, one major limitation of the PSO algorithm is the rate at which it reaches the optimal solution.

Further work in^13^ suggested a robust optimization(RO) strategy for attaining optimal scheduling of VPPs. A VPP with a Central Receiver Solar Thermal System(CRSTS), a Wind Turbine (WT), and an Electric Vehicle (EV) is the subject of^14^ investigation. The aim is to determine how well the Butterfly Optimization Algorithm (BOA) performs in mitigating system frequency and power deviations. This was done by employing BOA to prove its superior performance while adjusting the essential control parameters.

Further work in^15^ suggested a strategy to boost VPP's ability to use renewable energy, reduce its carbon emissions, generate revenues from carbon trading, and boost operating profits. The Compound Differential Evolution Algorithm (CDEA) is used in this case. Ref^16^ presents a synoptic control approach for a VPP in conjunction with metaheuristics. The method attempts to maximize power plant flexibility while supporting operational reserve needs, reducing CO_2_ emissions, and optimizing different components of VPPs to achieve specific goals. The various metaheuristics used are Simulated Annealing (SA), PSO, and Ant Colony Optimization (ACO). To minimize operational expenditure and maximize gains by reducing the need to purchase energy from an exterior source^17^, proposed a cross-entropy variable neighborhood differential PSO to assist the VPP in managing the available DERs as effectively as possible. The authors compared the proposed method to other optimization techniques. Towards a more sophisticated system^18^, suggested a proactive EM approach for an aircraft hybrid system made up of a proton membrane fuel cell, a battery, and a supercapacitor. The EM controls the states of charge of batteries and supercapacitors while reducing hydrogen consumption.

Metaheuristic optimization techniques provide strong tools for tackling stochastic optimization issues in VPP EM. They are highly suited for discovering robust and best solutions when stochastic elements are present. This is because of their ability to deal with uncertainty and explore huge solution areas. The performance of EM in VPPs can be improved by using the advantages of metaheuristics to address the difficulties posed by stochastic optimization. This has prompted numerous studies such as those elaborated earlier, and the ones summarized in Table 1. These studies aim at harnessing the advantages of metaheuristics to tackle the complexities of stochastic optimization in DER management.

**Table 1:** Selected literature on resources optimization.

Reference	Objective	Algorithm	Relevance
^26^	Optimal scheduling	HHO	Energy management
^14^	Power management	BOA	Voltage control
^27^	Carbon–neutral economic scheduling	MILP	Meeting climate target
^28^	Carbon emission reduction	SCV-PSO	Clean energy
^29^	Profit maximization	RO	VPP viability
^30^	Optimal scheduling	PSO	Power system flexibility
^31^	Emission reduction	BWO	Clean energy

VPPs as discussed earlier in this section represent a paradigm shift in the integration of DERs into the power grid, embodying the convergence of physical energy assets with sophisticated Information and Communication Technology (ICT) systems. This cyber-physical nature of VPPs introduces several cybersecurity challenges such as False Data Injection Attacks (FDIA), Denial-of-Service (DoS) attacks, necessitating a comprehensive understanding of their vulnerabilities and the evolving threat landscape.

FDIA represents a critical class of cyber threats that target the integrity of data in Cyber-Physical Systems (CPS), such as the VPP^19^. In FDIA, an attacker compromises sensor readings or control signals in a way that introduces errors into the system’s state estimation and decision-making processes^20^. The impact of FDIAs can be severe, as they can lead to incorrect operational decisions, system instabilities, and even catastrophic failures. The diversity of attack vectors and the complexity of CPS make FDIAs particularly challenging to detect and mitigate.

With this in sight, ensuring the security of the optimized generation data of the DERs at the Central Management System (CMS) node is the next step in the VPP's functioning following optimization. Precise and trustworthy data are essential to the best operation of VPP. The stability and effectiveness of the VPP may be affected by any departure from the system's actual state, which could result in less-than-ideal decisions being made. Achieving the expected benefits of VPP depends critically on supporting data integrity, which is not only a technical need but also a critical component.

Numerous documented cases spanning several years have highlighted the prevalence and impact of FDIA within power systems. These reported instances serve as compelling evidence of the persistent threat posed by FDIAs, underscoring the critical need for heightened vigilance, advanced detection mechanisms, and robust cybersecurity measures to safeguard the integrity and reliability of VPPs against such malicious acts. The 2010 discovery of the Stuxnet cyber virus, for example, was a sophisticated cyberweapon that was designed to attack Supervisory Control and Data Acquisition (SCADA) systems^21^. This weapon showed how malicious actors might alter data, threatening control system integrity and posing a serious risk to the dependable operation of vital infrastructure. Similarly to the Stuxnet cyber virus, a cyberattack caused significant disruption in the supply of energy in Ukraine in 2015, which caused a large power outage^22^. This unconducive situation occurred because hackers were able to corrupt the data integrity within the control systems, which resulted in the electricity infrastructure working incorrectly. Again, an attack on Saudi Aramco, the world’s largest oil company in 2012, experienced a significant cyberattack that affected 30,000 of its computers^23^. While the attack targeted oil production facilities rather than power systems directly, it serves as an example of the potential impact of FDIA on critical infrastructure. The attackers used malware to manipulate operations and cause widespread disruption. Although the specific details of data manipulation in this attack are not publicly disclosed, it highlights the vulnerability of industrial systems to cyber threats and the potential consequences of false data injection. It is important to note that, while these FDIAs are orchestrated by individuals, there are some that are also state sponsored. For instance, an attack known as the Dragonfly cyberattack targeted energy companies in the United States and Europe between 2011 and 2014^24^. The attackers, believed to be state-sponsored actors, infiltrated the networks of energy companies, and conducted preliminary investigations to gather information on critical infrastructure. While the primary objective was information gathering, it raised concerns about the potential for false data injection to disrupt energy systems. Although specific instances of false data injection in the Dragonfly attack have not been publicly reported, the incident underscores the on-going threat posed by cyber adversaries to power systems and critical infrastructure.

These actual incidents highlight the real-world dangers connected to data errors in infrastructure control systems, which undermines the reliability of critical infrastructure. In the VPP, where numerous DERs are aggregated and managed via CMS, the significance of data security is paramount. Ensuring the confidentiality, integrity, reliability, authenticity, accountability and availability of information and data is crucial to safeguarding VPP operations against cyber threats^25^. The possible consequences of compromised data go beyond the immediate operational difficulties and have the potential to harm the VPP’s resilience, sustainability, and economic viability in general. In this regard, tackling the issue of false data injection is necessary if VPPs are to be safeguarded against cyber threats.

Furthermore, another aspect of the VPP operation that is considered in this paper is the maximization of its profits in responding to grid demand via the energy market. In today’s electricity markets, VPPs are crucial actors. They actively take part in energy trading by aggregating and optimizing their DER portfolio in response to grid and market signals to profit from price conditions. VPPs participate in a range of energy markets to optimize the utilization of DERs and maximize revenue opportunities. These markets include the day ahead energy markets, the real-time energy markets, ancillary markets etc. Several documentations have been provided in literature to ascertain the profit maximization of VPPs. For example, in^32^^,^ a concept for using a bidding approach to optimize the VPP's profit while ensuring customer satisfaction was proposed. While the proposed approach aimed at developing an optimal bidding strategy for the VPP, the authors did not consider the effect of risk associated in the energy market. This limitation proves crucial as risks in the energy market can affect the revenue of the VPP. Furthermore, to increase the profits margin of numerous VPPs in the electricity markets^33^, proposed a double-stage model where the initial stage is for each VPP to maximize the outputs of its intrinsic units and the ability of its external units to interact with the neighboring VPP in the second stage. Engaging in the market for the day ahead^34^, analyzed the random nature of RESs and models a forecasting tool to reduce the ambiguity encountered with RESs generation to increase the marginal earnings of the VPP. This work considered the effects of RESs uncertainty which can prove to be a risk to affect the revenue stream of the VPP. However, while the authors looked at the transactions in the day-ahead market, it was imperative to consider a more comprehensive risk such as volatilities in pricing which greatly affects the revenue of the VPP. In optimizing the transactional judgement of pool market of a VPP, a scheduling strategy to optimize the generation contract of the VPP to improve the assets of the VPP is proposed in^35^. This strategy allows the VPP to strategically allocate its resources to generate and sell electricity at times when market prices are high. In another study, the optimal economic flow of a VPP is the subject of interest in^36^. In maximizing the power traded in the energy markets, the authors proposed a model to find the EM effectiveness by considering uncertainties of DERs and market prices.

Nevertheless, energy markets are not immune to risks, particularly those associated with price volatility, which can significantly impact VPPs operating within these markets. Price volatility refers to the rapid and unpredictable fluctuations in electricity prices due to various factors such as changes in demand, fuel prices, weather conditions, and market dynamics.

### Literature gap and contributions

The evolving landscape of the power grid, marked by deregulation, the rise of VPPs, integration of renewable energy sources, and growing environmental concerns, presents new challenges that need complex decision-making processes. These developments significantly expand the scope of decision variables, intensifying the complexity of power system management. Addressing these challenges demands tailored management strategies to optimize planning and operations effectively.

Optimization algorithms emerge as indispensable tools for decision-makers navigating the diverse variables inherent in power generation planning. Given the dynamic nature of the energy sector and the imperative to achieve Sustainable Development Goal 7 (SDG 7), continuous updates and enhancements to these algorithms are essential to align with evolving requirements and aims**.** This led to a reassessment of Pelican Optimization Algorithm’s (POA) architecture, a recently created algorithm that emulates the hunting behaviour of pelicans. POA is easy to apply because of its straightforward structure. However, because of the uneven distribution of exploration and exploitation, it is unable to give reliable performance. Thus, it was imperative to make the choice to investigate the effectiveness of an Improved Pelican Optimization Algorithm (IPOA) in addressing a range of operating assumptions and circumstances. This investigation comes after improvements meant to boost POA’s effectiveness and address the shortcomings.

Detecting data manipulation in VPPs poses distinctive challenges due to the decentralized and dynamic nature of VPP operations. These challenges stem from factors such as the diversity of energy sources, the need for real-time decision-making, and the high volume and velocity of data. Previous research has extensively examined anomaly detection techniques in power systems, exploring statistical and rule-based methods for identifying irregularities in data. However, recent advancements in machine learning have revolutionized false data detection, presenting a departure from traditional rule-based and statistical approaches. Machine learning algorithms, especially those designed for anomaly detection, offer the flexibility to adapt to the evolving characteristics of power systems. The control center of a VPP benefits from a wealth of data collected through wide-area measurement equipment, offering many opportunities for implementing advanced anomaly detection algorithms. Although several machine learning techniques have been employed to identify manipulated data in contemporary power systems, autoencoders offer a potent remedy for identifying data manipulation in a VPP setting, particularly in the context of time series data representations. Among these benefits are non-linearity and complexity handling, robustness to deviations and irregularities, unsupervised learning paradigm, among others. While classic autoencoders offer benefits such as non-linearity and complexity handling, convolutional autoencoders excel in capturing spatial and temporal patterns in time series data representations. In the context of identifying data manipulation in VPP settings, where temporal relationships are critical, convolutional autoencoders are better equipped to detect anomalies and deviations due to their ability to capture both spatial and temporal dependencies.

Also, the time-dependent nature of energy market fluctuations can be overlooked by conventional methodologies or models, which can oversimplify the complexities of price movements and result in poor risk management strategies and projections that are inaccurate. Time series forecasting is an approach based on past values of data to predict future values with the least predictable error. The Prophet application programming is better interpretable machine learning method in which non-linear trends are fit with yearly, weekly, and daily seasonality effects to fit the forecasting functions^37^. The choice of this tool lies in its adaptability for datasets that exhibit trends, seasonality, and holiday effects. VPP is advantaged to increase efficiency and profitability in the energy market by using Prophet to help make better decisions about pricing and risk management.

Thus, this paper presents a novel three-stage coordinated approach aimed at enhancing the reliability and efficiency of VPPs, addressing critical gaps in existing literature. Hence, the main contributions of this study are summarized as follows:

- Develop an energy management strategy for the VPP utilizing IPOA to optimally schedule DERs to meet grid demand. This stage of the coordinated approach contributes to the continuous improvement of optimization techniques for DER management within VPPs. Building upon the POA, IPOA, being an enhanced version is introduced. This advancement underscores the necessity of continuously refining optimization algorithms to achieve optimal results as evidenced in the results section of this paper.
- Develop a convolutional autoencoder model to learn normal patterns of the optimized data in meeting the grid demand. In contrast to earlier research, and to the best of the authors’ knowledge, much concentration has been given to cybersecurity in the context of the larger power system, which leaves cyber threats in VPP unattended to. The methodology employed includes a specific focus on data security in the VPP context. This is a major divergence from existing literature since VPPs are also susceptible to cyberattacks because they function in a highly networked digital environment.
- Employ the adaptive model in Prophet algorithm to mitigate price volatility risk in the energy market that the VPP participates in. This approach to mitigate price volatility in the energy markets offers VPPs an innovative and realistic solution to a major issue. The negative effects of price volatility on VPP revenue streams have frequently been overlooked, even though profit maximizing methods have been thoroughly studied in existing literature. This gap of overlooking the effects of price volatility risk is bridged by this paper by providing a proactive and data-driven method of managing price variations by integrating the Prophet model for day-ahead energy market price projections. The Prophet model is an effective tool for precisely projecting energy market prices because of its capacity to identify trend and seasonal components as well as its adaptability in managing non-linear patterns and outliers. As a result, VPP operators are better equipped to decide how best to participate in the energy markets and minimize risk by making well-informed choices about energy dispatch and trading.

Overall, this paper presents novel approaches to tackle major issues that VPPs encounter, such as pricing volatility, cybersecurity, and optimization efficiency. This paper makes a substantial contribution to the development of VPP technology and its role in influencing the direction of sustainable energy systems by filling in important gaps in the body of existing literature and providing realistic solutions.

**Figure 1:**
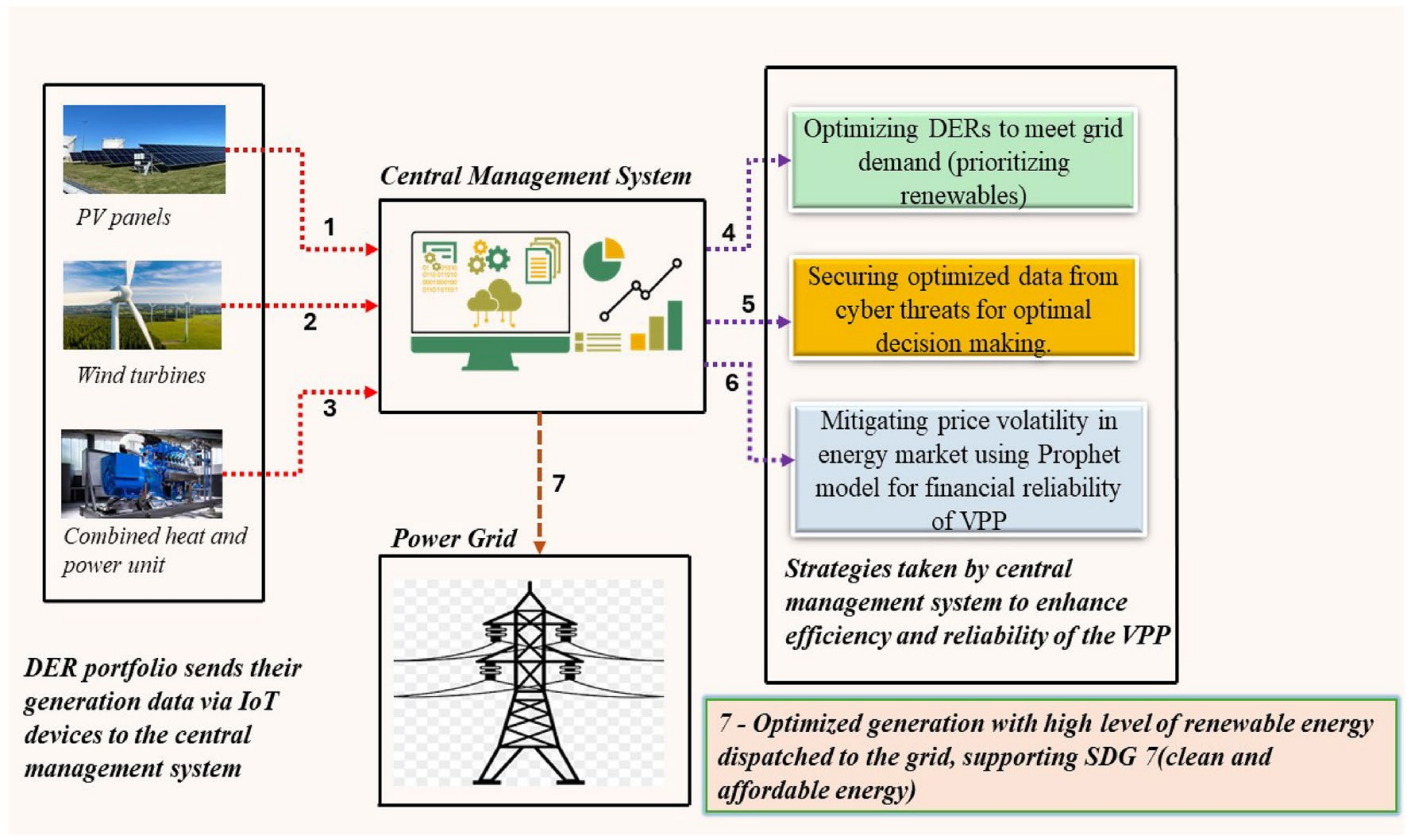
Block diagram of proposed approach.

The proposed approach for an efficient operational strategy of the VPP is illustrated in Fig. 1. The block diagram provides a holistic view of the processes involved in achieving the tri-level objective proposed in this paper. The rest of the paper is structured as follows. Section "VPP EM problem" presents the formulations of the approach. The techniques employed in solving the formulations are discussed in sections "Methods", "Results and discussion" presents the results, and the conclusions are provided in section "Conclusions".

## VPP EM problem

VPP EM problem encompasses the intricate task of optimizing the coordination and scheduling of DERs while considering generation costs and economic models. This multifaceted challenge involves deciding the most cost-effective and efficient use of diverse energy sources within the VPP. The best scheduling of DERs is pivotal in minimizing generation costs, maximizing revenue, and ensuring economic viability. Effectively addressing this challenge leads to not only improved economic performance but also enhanced overall operational efficiency and resilience of the VPP.

### Generation cost

The requested power can be shared across DERs in a multitude of ways that EMS encounters in practice. Minimizing the cost of power generation is the best approach. To do this, optimization is performed by considering a quadratic function for DERs within the VPP. Therefore, the operational function of generating unit $j$ can be explained as a quadratic function of the active power $\left({P}_{j}\right)$ given by Eq. (1)

1 $${C}_{j}\left({P}_{j}\right)= {a}_{j}{P}_{j}^{2}+ {b}_{j}{P}_{j}+ {c}_{j}$$

${C}_{j}$ depicts the cost of the power that each DER generates.

Power generated by individual DER is denoted by ${P}_{j}$

The constants that vary according to the kind of DER are a, b, and c.

An objective function f(x) is created based on the quadratic cost function for each DER in Eq. (2) to minimize the generation cost of the requested power. The aggregate of the generating costs for each DER determines the overall cost of energy production in the VPP.

2 $$Min {F}_{X}={\sum }_{j=1}^{NG}{a}_{j}{P}_{j}^{2}+ {b}_{j}{P}_{j}+ {c}_{j} j=1,\dots ,NG$$

In practical power systems, the cost function has non-derivative points due to the load or the wire-pull effect in thermal units. The thermal plant in this context of the VPP is replaced by the Combined Heat and Power (CHP) unit. This effect shows up as ripples in the fuel curve and makes the cost function a non-smooth, non-convex function. The effect is achieved by adding a rectified sinusoidal term, ${e}_{j}\text{sin}\left({f}_{j}\left({P}_{i}^{max}-{P}_{i}\right)\right)$ to the quadratic cost function as given in Eq. (3)

3 $$Min {F}_{X}={\sum }_{j=1}^{NG}{a}_{j}{P}_{j}^{2}+ {b}_{j}{P}_{j}+ {c}_{j}+||{e}_{j}\text{sin}\left({f}_{j}\left({P}_{i}^{max}-{P}_{i}\right)\right)||$$

The solver tries to iterate on the solutions until the ideal one is achieved after generating several initial feasible solutions. A challenging issue in optimization is how to address the equality requirement. The application of a penalty variable is one of the most popular solutions to this problem. Applying the penalty variable, the model becomes:

4 $$Min {F}_{X}={\sum }_{j=1}^{NG}{a}_{j}{P}_{j}^{2}+ {b}_{j}{P}_{j}+ {c}_{j}+||{e}_{j}\text{sin}\left({f}_{j}\left({P}_{i}^{max}-{P}_{i}\right)\right)|| +\text{C}f$$

In energy management model, penalty variable, which in this case in Eq. (4) denoted by $\text{C}f$ is used to express preferences and exert influence^38^. Its function is to steer the optimization process in the direction of solutions that meet the operational needs and intended results of minimizing the cost of production. In this paper, the optimization model is guaranteed to prioritize solutions that are in line with actual grid requirements by the penalty variable, which penalizes departures from precisely matching grid demand. This encourages operational accuracy, which supports dependable energy management tactics in the VPP.

### Constraints/technical limitation

Due to the DERs inherent variability, the optimization process is extremely a difficult one. To maximize the economic gains and contribute to a sustainable and robust energy system, it is imperative to address the optimization limitations or constraints (equality and inequality) to assure the best resource allocation and usage. The following constraints are taken into consideration to help the algorithm find the ideal value.

#### Power balance

In the energy management described in this paper, power balance acts as a constraint to make sure that the VPP total generation is equal to the real demand on the grid. The constraint, represented by the equality in Eq. (5) requires that the total power outputs of individual energy sources precisely meet the grid's consumption demand. This promotes grid stability and a dependable supply of energy. The power balance between the generated power of the DERs and the requested demand is given by:

5 $${\sum }_{j=1}^{NG}\left({P}_{j}\right)={P}_{L}$$

where ${\sum }_{j=1}^{NG}\left({P}_{j}\right)$ is the summation of power generated from the various DERs and ${P}_{L}$ is the requested demand.

#### Power generation limits

The permitted range of power outputs for each energy source inside the VPP is specified by the power generation limitations. These restrictions stop the energy sources from producing at levels below their minimal operating requirements or above their maximum ability. Equation (6) denotes the general notation of generation limits of an energy management problem.

6 $${P}_{min}\le {P}_{j} \le {P}_{max}$$

where ${P}_{min}$ and ${P}_{max}$ are the permissible power generation of each energy source respectively. Equation (6) implies that ${P}_{j}$ must be greater or equal to the minimum and less than or equal to the maximum values. This ensures that ${P}_{j}$ falls within the defined boundaries.

#### Ramp rate

The efficient management and integration of renewable energy sources is significantly influenced by ramp rate limits^39^. In this paper, ramp rate is defined as the rate at which the energy sources inside the VPP can alter their energy generation, that is, ramp up (increase generation) or ramp down (decrease generation). This parameter is essential for effective functioning since it affects the ability to control the variable energy output from renewable sources. Furthermore, ramp rate limit affects how the VPP's energy generation is scheduled and optimized, thus they must be considered while creating the best scheduling plans. The maximum and minimum permitted ramp-down (RD) and ramp-up (RU) values, as well as the prior power state ${P}^{o}$ of generating unit $j$, limit the unit’s output and prevent it from changing abruptly due to physical limits.

7 $${P}_{j}^{o}-{P}_{j}\le {RD}_{j}$$

8 $${P}_{j}-{P}_{j}^{o}\le {RU}_{j}$$

Since each generator's generating capacity is affected by the ramp rate restriction, the capacity constraints vary by Eq. (9).

9 $$Max\left({P}_{j}^{min},{P}_{j}^{o}-{RD}_{j}\right)\le {P}_{j}\le Min\left({P}_{j}^{max},{P}_{j}^{o}+{RU}_{j}\right)$$

#### Loss function

Losses serve as a critical constraint in the resource scheduling (energy management) to meet the grid demand. This constraint acknowledges the inherent inefficiencies in power transmission and emphasizes the need for the energy management model to account for these losses. The incorporation of loss constraint ensures a more exact representation of the energy flow dynamic within the VPP optimizing the balance between generation and delivery to enhance overall system performance. The mathematical model is provided below:

10 $${\sum }_{j=1}^{NG}\left({P}_{j}\right)={P}_{LS}+ {P}_{L}$$

where ${\sum }_{j=1}^{NG}\left({P}_{j}\right)$ is the summation of power generation from all the energy sources in the VPP,${P}_{LS}$ is the loss variable while the grid demand is denoted by ${P}_{L}$. These losses are brought on by factors such conductor quality, transmission distance, and line resistance.

In determining the losses, the Kron’s loss formula^40^, in Eq. (11), is used to calculate ${P}_{LS}$ in this paper. It approximates the loss function as a quadratic function of the power produced by the producing units.

11 $$P_{LS} = \mathop \sum \limits_{j = 1}^{NG} \mathop \sum \limits_{k = 1}^{NG} P_{j} B_{kj} P_{j} + \sum B_{ko} P_{j} + B_{oo}$$

In Eq. (11), ${B}_{kj}$ represents the loss coefficient that represent the bilinear terms in the Kron’s formula which captures the interaction between the power injections at different buses of the grid when the VPP dispatches its power to the grid. ${B}_{ko}$ is the loss coefficient that represents the linear term in the Kron’s formula which captures the relationship of power injection at a particular bus of the grid. ${B}_{oo}$ is a constant term which represents the fixed losses in the system independent of the power injections. This formula helps in planning the energy management of the VPP taking into account the transmission losses when it dispatches its power to the external grid as a response to the demand.

### Uncertainties of RERs

Uncertainties around the wind and photovoltaic farms in the VPP are critical factors to consider in energy management issues. Energy generation becomes unpredictable due to the intrinsic variability of these energy sources. Accurately predicting generation is a technique to oversee these uncertainties. This approach involves using forecasting models that consider meteorological data and historical performance to estimate the expected output of the renewable sources in the VPP.

#### Wind generation modelling

**Figure 2:**
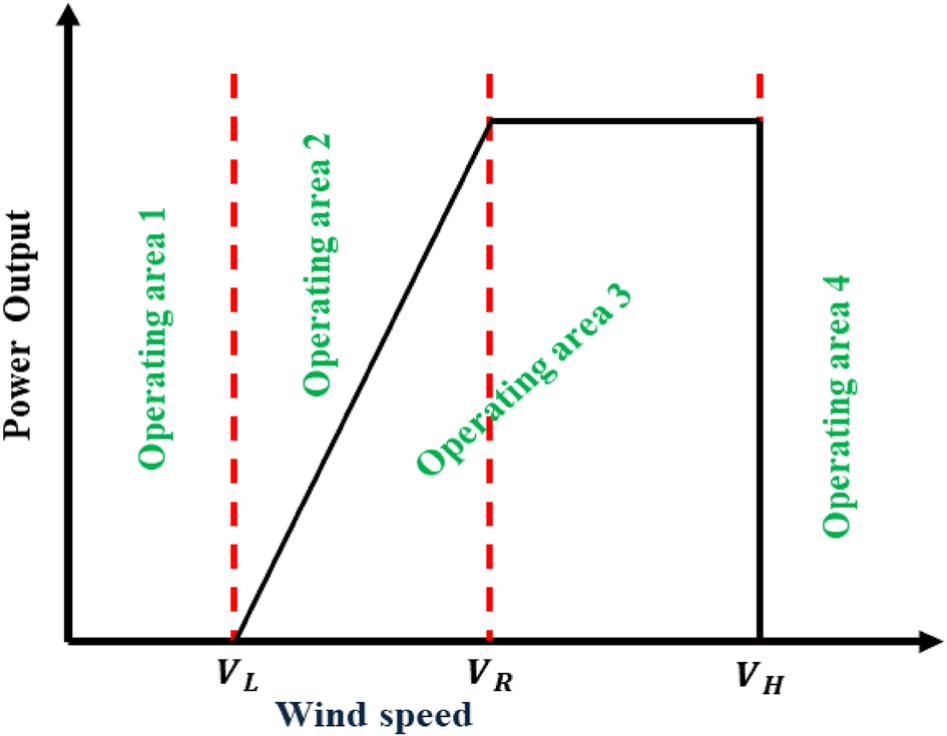
Operating areas of wind turbine.

Wind behaviour is stochastic and varies in direction and speed over time. This must be considered when modelling wind uncertainty. A probabilistic method such as the Weibull distribution is used to formulate a wind model that takes uncertainty into account.

12 $${f}_{V}\left(\upmu \right)=\left(\frac{m}{n}\right)\left(\frac{v}{n}\right)\text{exp}k-1 x {exp}^{-{\left(\frac{v}{n}\right)}^{k} },\upmu \ge 0$$

where ${f}_{V}\left(\upmu \right)$ is the probability distribution function (PDF) of wind speed *V* with scale parameter $m$, which is the point at which the PDF reaches its maximum and shape parameter $n$ which shows the degree of deviation of the observed distribution. Hence, the power produced by the wind turbine is calculated based on wind power curves provided by the manufacturer of the turbine as illustrated in Fig. 2, along with wind speed data pertinent to the site where the wind turbine is installed.Following the fundamentals of wind energy, the expected energy generated by the wind turbine can be computed as^41,42^ as given in Eq. (13).

13 $${P}_{w}^{t}=\left\{\begin{array}{c}0, \quad{V}_{(t)}<{V}_{L}, {V}_{(t)}>{V}_{H}\\ {(\eta }_{w}*{N}_{w}*{P}_{wr})*\frac{\left({V}_{\left(t\right)-}^{2}{V}_{L}^{2}\right)}{\left({V}_{R}^{2}-{V}_{L}^{2}\right)} , \quad{V}_{L}<{V}_{\left(t\right)}<{V}_{R} \\ {(\eta }_{w}*{N}_{w}*{P}_{wr}), \quad{V}_{R}<{V}_{\left(t\right)}<{V}_{H}\end{array}\right.$$

where ${P}_{w}^{t}$ is the output power of wind turbine, ${\eta }_{w}$ is the efficiency of the wind turbine, ${N}_{w}$ is the number of wind turbines with wind speed limits.

14 $${V}_{(t)}\ge {V}_{L}, wind turbine start to generate$$

where ${V}_{L}$ is the cut-in speed at which the wind turbine starts to generate.

15 $${V}_{H}>{V}_{(t)}, wind turbine stop generating$$

where ${V}_{H}$ is the cut-out speed at which the wind turbine stops generating to prevent damage to the turbine.

#### PV generation modelling

The power generated by each Photovoltaic (PV) farm depends on the number of PV panels $N$*,* the maximum output of each panel ${P}_{Panel}$, the efficiency of each panel $\eta$, and the sunlight intensity ${I}_{s}$ (solar irradiance)

16 $$P_{Pv} = N \cdot P_{Panel} \cdot \eta \cdot A_{Pv} \cdot I_{s}$$

Solar irradiance is also stochastic which requires a probabilistic method to be uncertainty. Rayleigh distribution is employed for the solar irradiance distribution which aids in the output generation forecasting of the PV farms.17where *F*(*Is; *б) is the PDF of solar irradiance with a scale parameter б which determines the variability of solar irradiance, and $Is$ is the solar irradiance data.

### Deterministic modeling of CHP

The CHP serves as a contingency solution within the VPP's DER portfolio, activated when the demand from the grid exceeds the ability of PV and wind turbines. As a conventional energy source, its role is to support uninterrupted and dependable power supply by compensating for any shortfall between demand and the output from the RERs. Equation (17a) can be used to determine the hourly fuel consumption of the CHP^43^.

17a $${X}_{c}^{t}=\left(Y*{P}_{hj}^{t}\right)+\left(Z*{P}_{{r}_{chp}}\right)$$

where ${X}_{c}^{t}, {P}_{hj}^{t}, {P}_{{r}_{chp}}$ are fuel consumption, hourly generated power output, and rated power of CHP, respectively. The constant parameters Y and Z have an estimated typical value of 0.246 and 0.8415, respectively.

### VPP data security formulation

An attack on a VPP's state estimator entails tampering with measurements and data to falsify the state estimation procedure. Importantly, the state estimator oversees the system's current state using the data from the optimization process. The VPP’s profile is determined by using data collected from the optimization process through end devices, such as meters, sensors, relays, etc. After that, this data is used for purposes such as satisfying grid demand, as it is the aim of this study. If these readings are fake, the VPP will make poor decisions because of inaccurate assessments of the system’s state.

Estimating the current condition of the system, including monitoring and forecasting, is usually the responsibility of the CMS in a VPP. Like the state estimator in the external power grid, the CMS in a VPP performs similar functions. A VPP's CMS uses several models and algorithms to track and predict how well DERs are performing inside the VPP. To evaluate the status of the energy system for the best possible decision-making and operation, it examines historical trends, real-time data, and other pertinent information.

Consider the VPP with p state variables represented by the vector, y. The measurements, N, can be related to the state variables through a linear measurement model in Eq. (18).

18 $$N=Hy+V$$

where $N$ is the vector measurement, $H$ is the Jacobian matrix, $V$ is the vector measurement errors, covariance matrix R, and mean zero, which are assumed to be normally distributed.

19 $$H\left( x \right) = \frac{1}{2}\mathop \sum \limits_{i = 1}^{m} W_{i} (\tilde{N} - N)^{2}$$

The weighted residuals show the discrepancies between the estimated values and data inflow about the system variables. These weighted residuals are essential. The purpose of employing weighted residuals is to provide a more exact and dependable estimate by assigning greater weight to measurements with lower variances. The weighted residuals are determined mathematically in the following way:

20 $$x={H}_{y}-N$$

where the vector of measurement is N, the vector of the state variables to be estimated is y, the Jacobian matrix is H, and the vector of weighted residuals is x. Upon obtaining the residuals, the system's state is determined by reducing the weighted total of the squared residuals. Mathematically, the solution is represented as:

21 $$\hat{X} = \arg \min_{x} \left( {H\left( y \right) = \frac{1}{2}\mathop \sum \limits_{i = 1}^{m} W_{i} (\tilde{N} - N)^{2} } \right)$$

The introduction of false data shown in Eq. (22) is as a of malicious attempts from hackers who intentionally manipulate the optimized data to compromise the VPP’s state estimation.

22 $$MD=GD+AV$$

where MD is the manipulated data to be injected by the attacker. This manipulated data constitutes genuine data or the optimized data GD, at the CMS of the VPP sent by the measuring and sensing units, and the attacking vector, AV. The AV is based mostly on statistical concepts, providing a strong framework for expressing the joint probability distribution of several variables in the VPP. The AV in this study is constructed using the Gaussian Distribution (GD)^44^, with probability distribution in Eq. (23), with $\mu$ ss the mean, which represents the central location of the distribution, $\sigma$ as the standard deviation which determines the spread or dispersion of the distribution

23 $$f\left( {N|\mu ,\sigma } \right) = \frac{1}{{\sigma \sqrt {2n} }}e^{{ - \frac{{(X - \mu )^{2} }}{{2\sigma^{2} }}}}$$

#### Data preprocessing of optimized generation data

In the preprocessing of the optimized data of the VPP, several key steps are undertaken. Firstly, normalization is applied to ensure consistent feature scales across the dataset. Feature engineering is then employed to select or transform features that effectively capture operational characteristics. Temporal segmentation is conducted to divide the data into meaningful intervals, enabling the detection of patterns within the data. A validation split is performed to distribute a portion of the data for model validation and performance monitoring. Finally, sequence padding ensures uniform sequence lengths across the dataset, facilitating model training and analysis.

### The economic modeling

The process of examining the activities of the electricity market where the VPP sells its generated capacity is part of the economic modeling. The models presented make it easier to understand and optimize a variety of pool-related factors, including supply–demand dynamics, markets risks and risk management strategies, helping the VPP to accomplish its goals. In this paper, the energy market considered is the day-ahead energy market.

Energy transactions in the day-ahead market represent a pivotal aspect of electricity trading, providing a forward—looking mechanism for the VPP to meet the grid demand. Consider the revenue of the VPP, the corresponding mathematical model is formulated as follows^45^.

24 $$Max Profit=max\sum_{i=1}^{ND}{P}_{F}=\sum_{i=1}^{ND}{R}_{V}-\sum_{j=1}^{NG}{C}_{j}({P}_{j})-\sum_{k=1}^{MR}{{P}_{R}}^{assesment}$$

where ${P}_{F}$ is the profit the VPP makes after selling its generated power to the grid, ${R}_{V}$ is the revenue from selling the power, ${C}_{j}({P}_{j})$ is the cost of producing the power, and ${P}_{R}$ is the price volatility risk in the energy market. Price fluctuation should be considered a risk factor in the VPP's evaluation, even though it generates profit from selling power to the grid through the energy market.

With the knowledge from the optimization, the VPP knows how much energy it can produce in the coming day and communication is done between the VPP operator and the external grid via the Day Ahead Market (DAM) operator, specifying how much electricity it will supply and how much it will sell it for to the grid. Thus, the revenue generated by the VPP is formulated as^46^. The DAM is considered in this paper.

25 $$\sum_{i=1}^{ND}{R}_{V}=\sum_{j=1}^{NG}{P}_{j}*spot \,price\, in\, DAM$$

Risk management is crucial from the standpoint of the VPP, especially considering the dynamic nature of energy markets and the potential negative impact on revenue streams. In this paper, the Conditional Value at Risk (CvaR) measurement in the DAM is introduced^45^. The CVaR is introduced to assess the DAM risk. Afterwards, mitigation of the risk is conducted.

26 $$\sum_{k=1}^{MR}{{P}_{R}}^{assesment}={CVaR}_{\alpha }=\left[E\left|X\right|>{VaR}_{\alpha }\right]=\left(\frac{1}{1-\alpha }\right){\int }_{\alpha }^{1}{F}^{exp-1}\left(t\right) dt$$

where ${F}^{exp-1}$ is the inverse cumulative distribution function of the loss distribution, and $\alpha$ is the confidence level and $VaR$ provides an estimate of the maximum loss that the VPP incur within a given level over a certain period. It is given as^47^.

27 $${VaR}_{\alpha }=\text{inf}\left\{X\epsilon {\mathbb{R}}:P\left(X\le x\right)\ge 1-\alpha \right\}, for\, \alpha \,confidence\, level$$

With the use of CVaR, financial risks in the economic modelling of VPPs can be effectively assessed. This improves the resilience and overall financial performance of VPP operations while aiding VPP operator in making well-informed decisions on price volatility mitigation. After the assessment of the financial risk, the VPP operator employs the Prophet model, which is discussed in later section of this paper to mitigate the risk of price volatility in DAM. With this in place, VPP operators can improve the resilience and financial performance of their operations, enhancing the sustainability and competitiveness of the VPP in the energy market.

## Methods

This section provides a concise overview of the existing challenges associated with POA and underscores the need for improvement. It introduces the proposed algorithm (IPOA) designed for scheduling DERs to meet the demand of the external grid as a potential solution. Additionally, the section delves into the significance of early detection of manipulated optimized data within VPPs, emphasizing the importance of data integrity. Finally, it presents a strategy aimed at mitigating price volatility, empowering VPPs to conduct robust economic analyses.

### Overview of POA

The POA is a recently created swarm-based metaheuristic algorithm that draws inspiration from nature, specifically from the hunting habits of pelicans^48^. The three primary phases of the POA are as follows:

Moving toward prey (exploration phase): During this phase, pelicans find the prey, which is typically a flock of fish. They then use Eq. (28) to move in the direction of the prey to get a better position and dive in.

28 $${X}_{j}=LB+rand*\left(UB-LB\right), j=\text{1,2},3,\dots ,n$$

where n is the number of populations, ${X}_{j}$ is the value of the candidate solution, and rand is the random vector interval. The optimization goal for the proposed issue is evaluated using the solutions to those initial candidates. The vector of the objective function is then determined. To update the pelicans’ hunting activity is mimicked as they attack their food source.Approaching Food Source (Investigating phase): This stage mimics pelican’s method of identifying food sources by scanning the area. The Pelicans locate their target and begin to move in their direction. One crucial aspect of the prey site is generated at random by POA, boosting exploring power. The *j*th pelican candidate solution's new state is found using mathematical representation.

29 $${X}_{j}^{new}=\{{X}_{j\left(t\right)}+rand*\left({X}_{j}-I\right), F\left({X}_{p}\right)\le F\left({X}_{j}\right){X}_{j\left(t\right)}-rand\left({X}_{p}-I.{X}_{j}\right)$$

where ${X}_{p}$ is the randomly created position of the prey, $I$ is a randomly generated vector with a value of 1 or 2, and $F\left({X}_{p}\right)$ is the value of its objective function. The solution is then updated by the new position as follows:

30 $${X}_{j\left(t+1\right)}=\{{X}_{j}^{new}, F({X}_{j}^{new})\le F({X}_{j}){X}_{j\left(t\right)}$$

Winging on the water surface (exploitation phase): Pelicans start to spread their wings on the water's surface at this point to allow the prey to ascend. The updated state of the *j*th pelican competitor solution in this stage is modeled as:

31 $${X}_{j}^{ne{w}_{2}}={X}_{j\left(t\right)}+Q.\left(\frac{1-t}{{M}_{max}}\right)*\left(2.rand-1\right)$$

where $Q$ is a constant equal to 0.2, and t is the current iteration. After that, the solution is updated based on the new position as:

32 $${X}_{j\left(t+1\right)}=\{{X}_{j}^{ne{w}_{2}}, F({X}_{i}^{ne{w}_{2}})\le F({X}_{j}){X}_{j\left(t\right)}$$

#### Shortcomings of POA

The POA converges quickly and has a straightforward structure. However, this haste could cause it to get stuck in local optima, which would impair performance for the reasons.

- The current solution depends only on the information provided by the random target and does not personally know the target's position, as per Eq. (28). The shift is dependent on the fitness value of a random target. Shifting is beneficial when the random target is either travelling in the unknown direction of the best solution or is itself the best solution. It does, however, offer a great ability for exploration.
- Eq. (31) suggests that the exploitation phase is equivalent to the idea of a mutation with a time-dependent, linearly reducing neighborhood radius $Q$. Updates to the present solution are insensitive to the nearby solutions and the size of the mutation, even though the neighborhood radius is considered. Thus, its value is the only factor affecting the current solution. so that the likelihood of settling in the local optima rises and the variety falls. Navigating these local optima may therefore benefit from taking adjacent reactions impacts into account.

### Improved POA

Updates and improvements of the generic POA are provided in this subsection.

#### Movement strategies

Because of their swarm intelligence, pelicans often hunt in groups. To obtain a better position and raise their success rate in obtaining food, members of this bird flock compete with one another, much like in other bird flocks.

Three distinct techniques considering this.

- The first technique is the fitness of the pelicans. If $F(\overrightarrow{{X}_{j}^{k}})<F(\overrightarrow{{X}_{i}^{k}})$ is satisfied, it shows that one of Eqns. (34), (36) and (38) are preferred by the present pelican and thus the position of the random pelican is better. If not, the movement is prioritized by the random pelican, $\overrightarrow{{X}_{j}^{k}}$ which updates its location by moving closer to the current pelican $\overrightarrow{{X}_{i}^{k}}$ by selecting one of the movement strategies based on Eqns. (35), (37) and (39).
- The second option is the self-knowledge movement strategy, where the pelican uses its own knowledge to decide where to go next in relation to the food source and uses Eq. (36) to determine when, ${q}_{1}<{F}_{CK}$ is fulfilled, even though the randomly picked member is more fit. When the requirement of, ${q}_{1}<{F}_{CK}$ is not met, the pelican grows pessimistic and prefers to rely on group knowledge.
- The next phase is the member-based movement, in which the pelican only depends on a randomly selected group member to determine its movements if the amount of knowledge is smaller than its intended quota ${q}_{1}<{F}_{CK}$ according to Eq. (36). The random pelican uses ${q}_{2}<{F}_{CK}$ and Eq. (37) to set up its position. If not, the group-based movement tactic will be used.
  33 $$\overrightarrow{{X}_{j,d}^{t+1}}=\overrightarrow{{X}_{j,d}^{t}}+\overrightarrow{{q}_{3}} .(\overrightarrow{{X}_{best}^{t}}-I. \overrightarrow{{X}_{j,d}})$$
  34 $$\overrightarrow{{X}_{j,d}^{t+1}}=\overrightarrow{{X}_{j,d}^{t}}+\overrightarrow{{q}_{3}} .(\overrightarrow{{X}_{best}^{t}}-I. \overrightarrow{{X}_{i,d}})$$
  35 $$\overrightarrow{{X}_{j}^{t+1}}=\overrightarrow{{X}_{j}^{t}}+\overrightarrow{{q}_{4}} .(\overrightarrow{{X}_{i}^{t}}-I. \overrightarrow{{X}_{j}})$$
  36 $$\overrightarrow{{X}_{j}^{t+1}}=\overrightarrow{{X}_{j}^{t}}+\overrightarrow{{q}_{4}} .(\overrightarrow{{X}_{j}^{t}}-I. \overrightarrow{{X}_{i}})$$
  37 $$\overrightarrow{{X}_{j}^{t+1}}=\overrightarrow{{X}_{j}^{t}}+\overrightarrow{{q}_{5}} . \left(\overrightarrow{{X}_{i}^{t}}-I. \overrightarrow{{X}_{j}^{t}}\right)+\overrightarrow{{q}_{6}} . \left(2.\overrightarrow{{q}_{7}} . \overrightarrow{{X}_{best}^{t}}-I. \overrightarrow{{X}_{j}^{t}}\right)$$
  38 $$\overrightarrow{{X}_{j}^{t+1}}=\overrightarrow{{X}_{j}^{t}}+\overrightarrow{{q}_{5}} . \left(\overrightarrow{{X}_{j}^{t}}-I. \overrightarrow{{X}_{i}^{t}}\right)+\overrightarrow{{q}_{6}} . \left(2.\overrightarrow{{q}_{7}} . \overrightarrow{{X}_{best}^{t}}-I. \overrightarrow{{X}_{i}^{t}}\right)$$

Note that $\overrightarrow{{q}_{j}}$ is a scalar value and take random values from the uniform distribution. Movement techniques have the potential to drive newly determined positions beyond acceptable bounds in some dimensions. Equation (38) is therefore used to rectify the violation. Next, based on an evaluation of the potential solution, the replacement is implemented via optimistic choice according to Eq. (39).

39 $$\overrightarrow{{X}_{j,d}^{t}}=f\left(x\right)=f\left(x\right)=\left\{\text{max}(\overrightarrow{{X}_{j,d}^{t}}\right., \overrightarrow{{LB}_{j}^{t}})\text{min}\left(\overrightarrow{{X}_{j,d}^{t}}, \overrightarrow{{UB}_{j}^{t}}\right)$$

#### Winging phase

**Figure 3:**
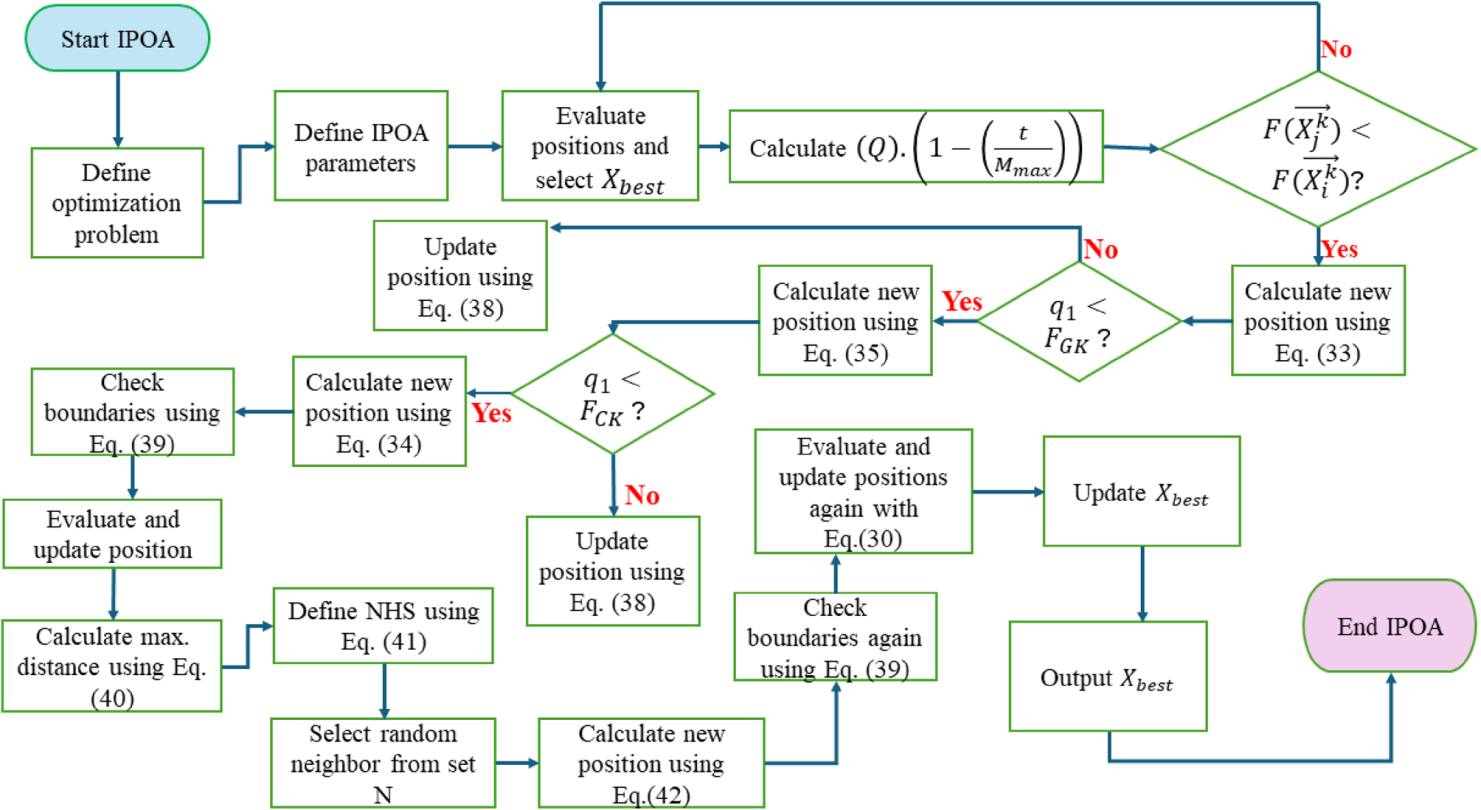
IPOA flowchart.

**Figure 4:**
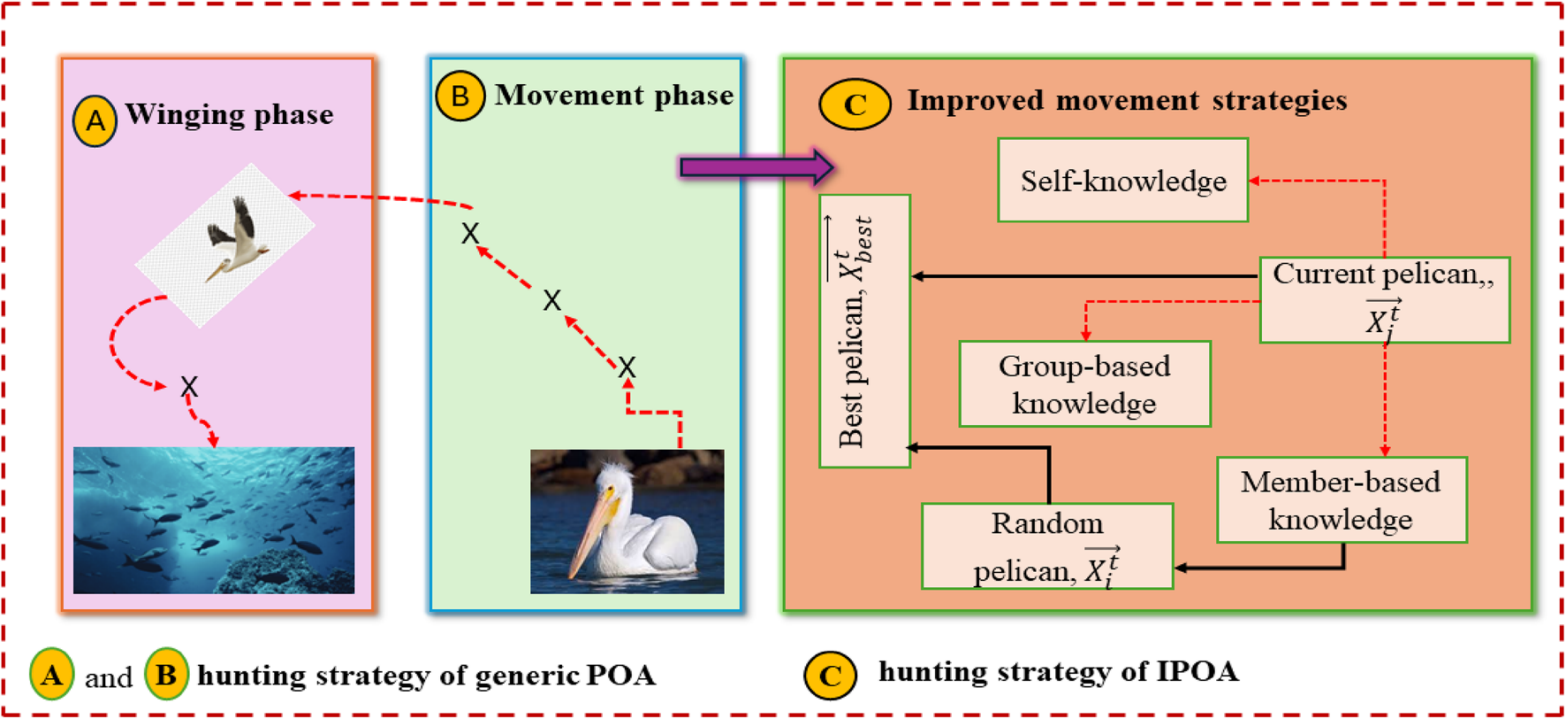
Hunting strategy of POA and IPOA.

The mutation is conducted using the dimension learning based technique once all individuals have been relocated. To use this strategy, the largest radius of the Neighborhood Set (NHS) is first computed using Eq. (40) which calculates the distance between current best solution and other solutions in the solution space, and then randomly determines the total number of dimensions for the mutation. Next, for Eq. (41), every individual within this range is part of the neighborhood set which calculates the set of neighboring solutions within a certain distance. Following the creation of the neighborhood set, the randomly picked neighbor's random dimensions are subjected to the dynamic mutation rule, as per Eq. (42) improves solutions over multiple iterations. The procedural steps with the IPOA are illustrated by the flowchart in Fig. 3. Also, the hunting strategy of POA and the IPOA are illustrated in Fig. 4.

40 $${E}_{max}^{t}=\left|\left|\overrightarrow{{X}_{best}^{t}}-\overrightarrow{{X}_{j}^{t}}\right|\right|$$

41 $${NHS}_{j}^{t}=(\overrightarrow{{X}_{N}^{t}}\left|\left|\overrightarrow{{X}_{j}^{t}}-\overrightarrow{{X}_{N}^{t}}\right|\right|\le {E}_{max}, N=1,\dots ,{N}_{p}$$

42 $$\overrightarrow{{X}_{j,d}^{t+1}}=\overrightarrow{{X}_{j,d}^{t}}+\left(Q\right).\left(1-\left(\frac{t}{{M}_{max}}\right)\right).\left(2.rand-1\right).\left(\overrightarrow{{X}_{j,d}^{t}}-\overrightarrow{{X}_{N,d}^{t}}\right)$$

### Convolutional autoencoder for data manipulation detection

**Figure 5:**
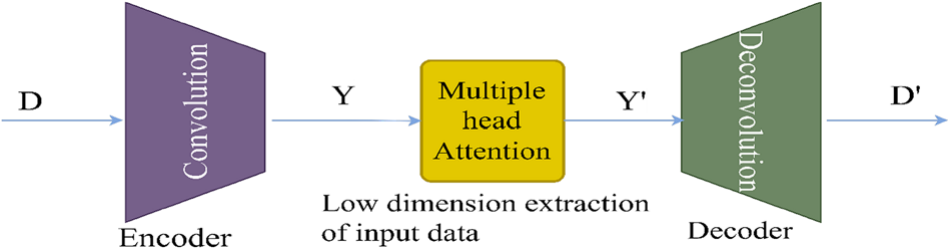
Architecture of Convolutional Autoencoder.

A convolutional autoencoder is a neural network architecture used for unsupervised learning. It consists of an encoder-decoder structure where convolutional layers are employed to extract features and learn representations of input data^49^. In the context of VPPs, the convolutional autoencoders will play a crucial role in detecting data manipulation. As VPPs get more complicated and decision-making becomes more dependent on data, it is critical to guarantee the accuracy and consistency of data. Within VPP datasets, convolutional autoencoders provide a reliable way to find abnormalities and evidence of data manipulation^50^. The convolutional autoencoder illustrated in Fig. 5 is an efficient way to learn the underlying distribution of normal operational data by taking advantage of the natural spatial structure and patterns found in the data.

The process of encoding the data D from the output layer to the hidden layer:

43 $$D=Encoder\left(D\right)$$

The procedure for obtaining low-dimensional features from the data:

44 $${D}{\prime}=MHA\left(Y,Y,Y\right)$$

where MHA is the multiple head attention representation of the convolutional autoencoder which enables the model to focus on various parts of the input data simultaneously.

The decoding process from the hidden layer to the output layer:

45 $${Y}{\prime}=Decoder\left({D}{\prime}\right)$$

Encoding the high-dimensional input, $D$ into a low-dimensional latent variable $Y$ is the encoder's function. This allows the neural network to notice the most informative aspects. Additionally, the decoder's function is to rebuild the low-dimensional spatial characteristics ${Y}{\prime}$ and transfer the essential information back to the original input space. Transposing convolutional layers to enhance width and height allows for the transformation of a narrow representation into a wide reconstruction matrix, which is what decoding entails. These layers work on an identical concept to convolutional layers, albeit in reverse. The activation function for the convolutional autoencoder utilized is the Rectified Linear Unit (ReLU). The hyperparameters used in the convolutional autoencoder as shown in Table 2 aided in the model’s ability to learn efficiently the meaningful representations from the optimized generation data from the DERs.

**Table 2:** Hyperparameters setting of convolutional autoencoder.

Hyperparameter	Value
Encoding dimension	12
Learning rate	0.009
Batch size	64
Epochs	100

### Economic risk mitigation using prophet model

Economic Risk Mitigation using the Prophet Model in VPPs involves employing advanced forecasting techniques to predict and manage potential price volatility in the energy market. The Prophet Model, known for its robustness in time series forecasting, offers a valuable tool for the VPP operator to mitigate economic risks^37^. The integration of the Prophet Model for economic risk mitigation in the VPP is a significant step towards enhancing the economic viability and sustainability of the system. Utilizing the model, VPP operators can more skillfully negotiate energy market price uncertainty and maximize revenue streams within dynamic energy markets.

Mathematically, the model is presented as follows^51^:

46 $$Y_{x} = G_{x} + S_{x} + H_{x} + \varepsilon_{x}$$

where ${G}_{x}$ is the observed DA market prices of the time series at time *x,* ${S}_{x}$ represents the seasonality component, ${H}_{x}$ represents the holiday effect and ${\varepsilon }_{x}$ is the error term, capturing any remaining variability not accounted for.

The seasonality in the model refers to the repetitive and predictable patterns that occur at regular intervals over time. The patterns used in this model correspond to daily intervals. Seasonality manifests as regular fluctuations or variations in the data, and it plays a crucial role in understanding and forecasting patterns. The seasonality part, ${S}_{x}$ is given as follows.

47 $$S_{x} = \mathop \sum \limits_{i = 1}^{Nseason} a_{i} \sin \left( {\frac{{2\pi \left( {t - \phi_{i} } \right)}}{{P_{i} }}} \right) + \left( {b_{i} \sin \left( {\frac{{2\pi \left( {t - \phi_{i} } \right)}}{{P_{i} }}} \right)} \right)$$

where $Nseason$ is the number of seasonal components, ${P}_{i}$ is the period of the *i*th seasonal component, *φ*_*i*_ is the phase of the *i*th seasonal component, ${a}_{i}$ and ${b}_{i}$ are the amplitude parameters. The following drives the reason the Prophet time series model was chosen over other algorithms.

- Automatic seasonality detection
- Robustness to missing data and outliers.
- Scalability
- Flexibility

Historical price data from the Nord pool^52^ energy market was considered for the predictive analysis. The market analysis considered for this study is the day-ahead energy market (DAM).

## Results and discussion

This section presents the results and discussions of the processes taken in ensuring efficient operation of the VPP. Firstly, optimization is performed to optimally schedule the DERs to meet the grid demand at the lowest cost possible. The outcome of the scheduling using the IPOA is compared with POA, GA, and PSO. In the second stage, the results in using the convolutional autoencoder to secure the optimized generational data from cyber threats are also presented. The result at the second stage is also compared with the traditional autoencoder model, and other machine learning algorithms. Lastly, economic analysis is done to find the financial viability of the VPP in responding to the grid demands by trading its generated power via the day-ahead energy market.

### Optimal scheduling of DERs by the VPP.

The grid-initiated demand response by the VPP signifies a dynamic and responsive approach to grid management by actively engaging with the grid’s signals. This approach positions the VPP as a valuable partner in enhancing overall grid reliability, resilience, and efficiency while simultaneously perfecting its own economic performance.

**Figure 6:**
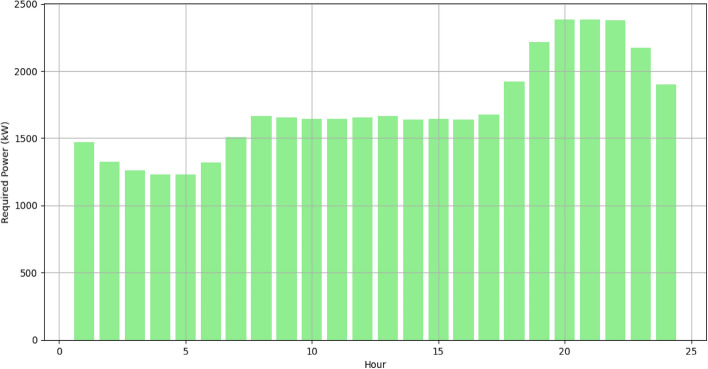
Grid power demand.

To evaluate the efficiency of the approach, optimization is performed with different algorithms using a system topology where the demand location is an IEEE 37 node test feeder^53^ through various case studies. In this paper, the portfolio of DERs of the VPP consists of three wind farms with capacity of 750kW each, two photovoltaics farms with a capacity of 200kW each, and a CHP unit with a capacity of 1000kW. The required power from the grid at each hour is illustrated in Fig. 6. The optimization is done with MATLAB software (Version number: 2023a, URL link: https://in.mathworks.com/products/new_products/release2023a.html) on a computer with core i7 processor and an 8GB memory. The algorithms are both set to 20 independent runs to find their respective ideal or best solutions. The computational efficiency of the optimization process shows the superiority of IPOA to other algorithms in achieving the objective function set in section "Generation cost" Eq. (1) with relatively lower computational time as shown in Table 3.

**Table 3:** Algorithm performance comparison.

Algorithm	Number of iterations	Search agents	Number of runs	Execution time (s)
GA	100	50	20	131
PSO	100	50	20	128
POA	100	50	20	125
IPOA (proposed)	100	50	20	116

With respect to the algorithms solving the same objective function (minimizing production cost while meeting demand) with the same parameters, the lower execution time of IPOA indicates that it achieves better convergence to the optimal solution. On the other hand, longer computational time delays decision-making, which leads to suboptimal solutions. It also hinders algorithm exploration and convergence, limiting optimal solutions. This makes IPOA preferable when computational efficiency is a priority, as it can achieve better results in less time compared to the other algorithms. This computational efficiency of IPOA in optimizing the scheduling of the DERs plays a crucial role in enhancing the VPP’s performance in market time horizons when trading. It enables faster decision-making, ultimately driving the VPP towards greater success in energy markets.

**Figure 7:**
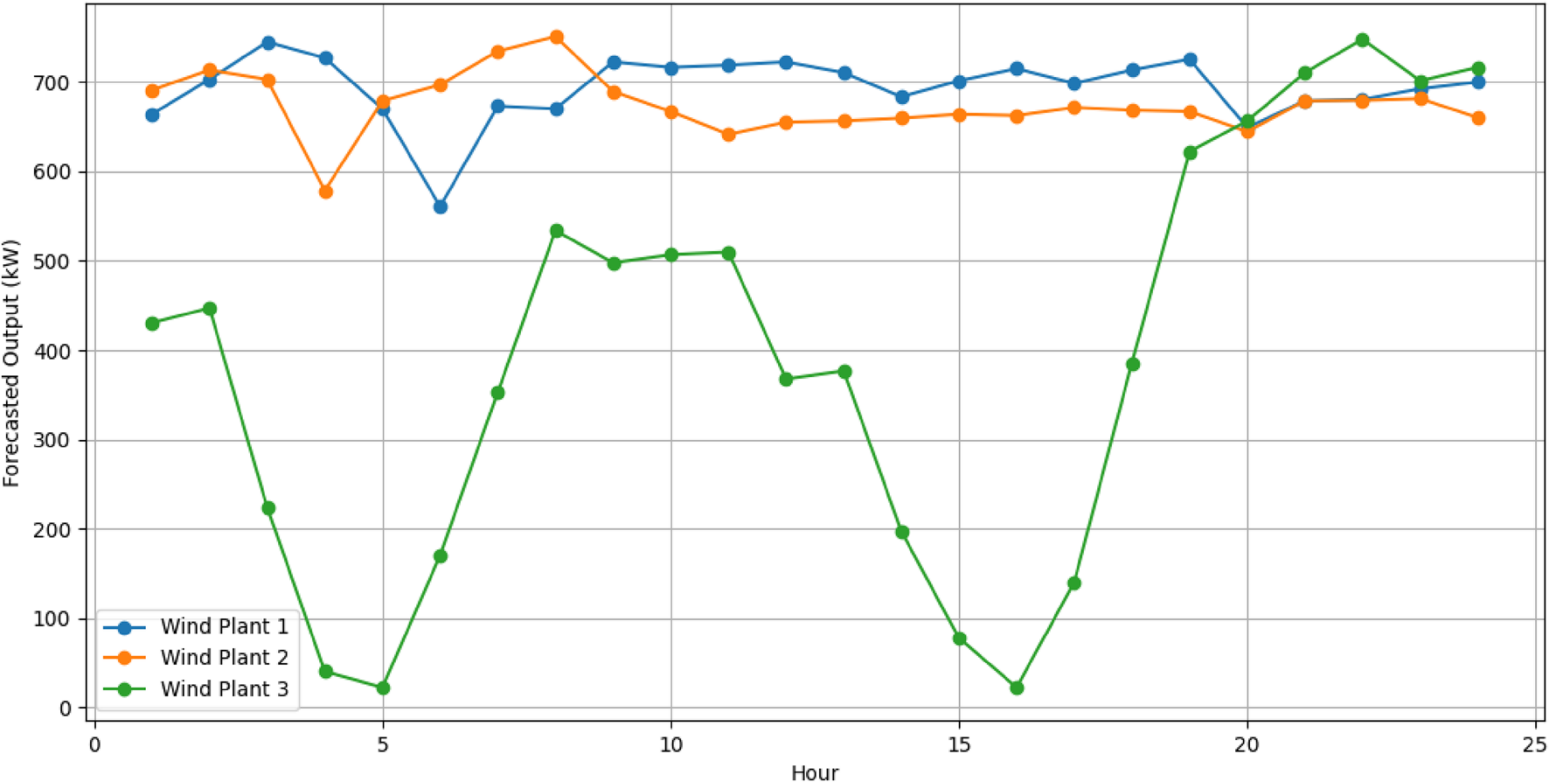
Forecasted output of wind plants.

**Figure 8:**
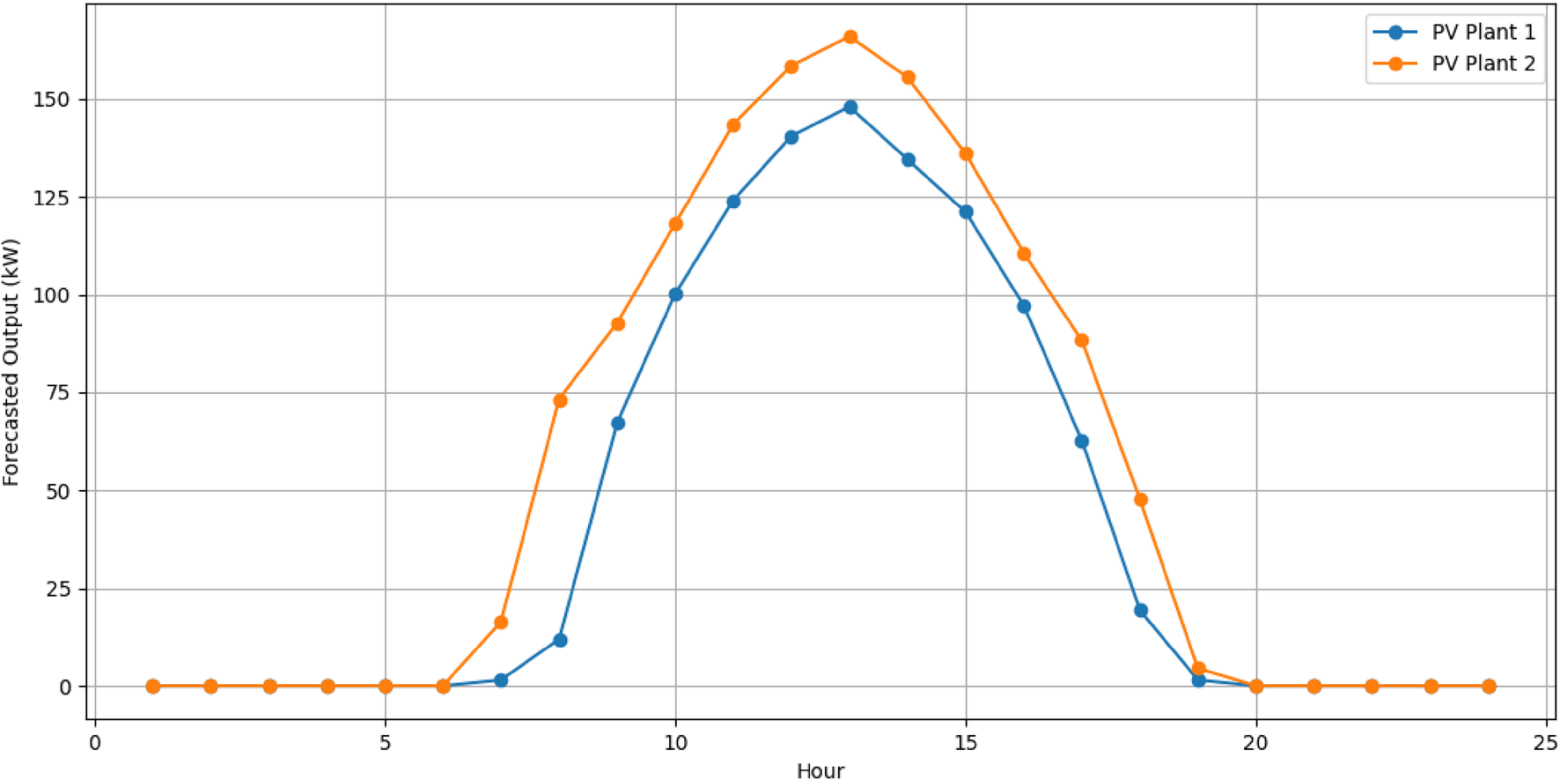
Forecasted output of PV plants.

The RERs power generation forecast is made and shown in Figs.7 and 8. Forecasting wind and solar generations in the VPP is a pivotal part for effective resource scheduling. Accurate forecasts of the RERs empower the VPP to proactively adjust the demand from the DERs in alignment with expected fluctuations in renewable energy output. This precision allows the VPP to strategically harness excess renewable energy during periods of high generation, optimizing resource use and minimizing reliance on the CHP.

#### Case studies

Three different case studies with different VPP configurations, presented in Table 4 are considered for the optimization of the DERs to meet the grid demand. The results obtained from IPOA are contrasted with those of the generic POA, GA and PSO to assess the performance of the proposed algorithm.

A.A. Case study A

**Table 4:** Case studies with associated configurations.

Case study	Configuration
A	All generators are working
B	PV A is not generating, the rest are working
C	CHP is isolated, the rest are in operation

**Figure 9:**
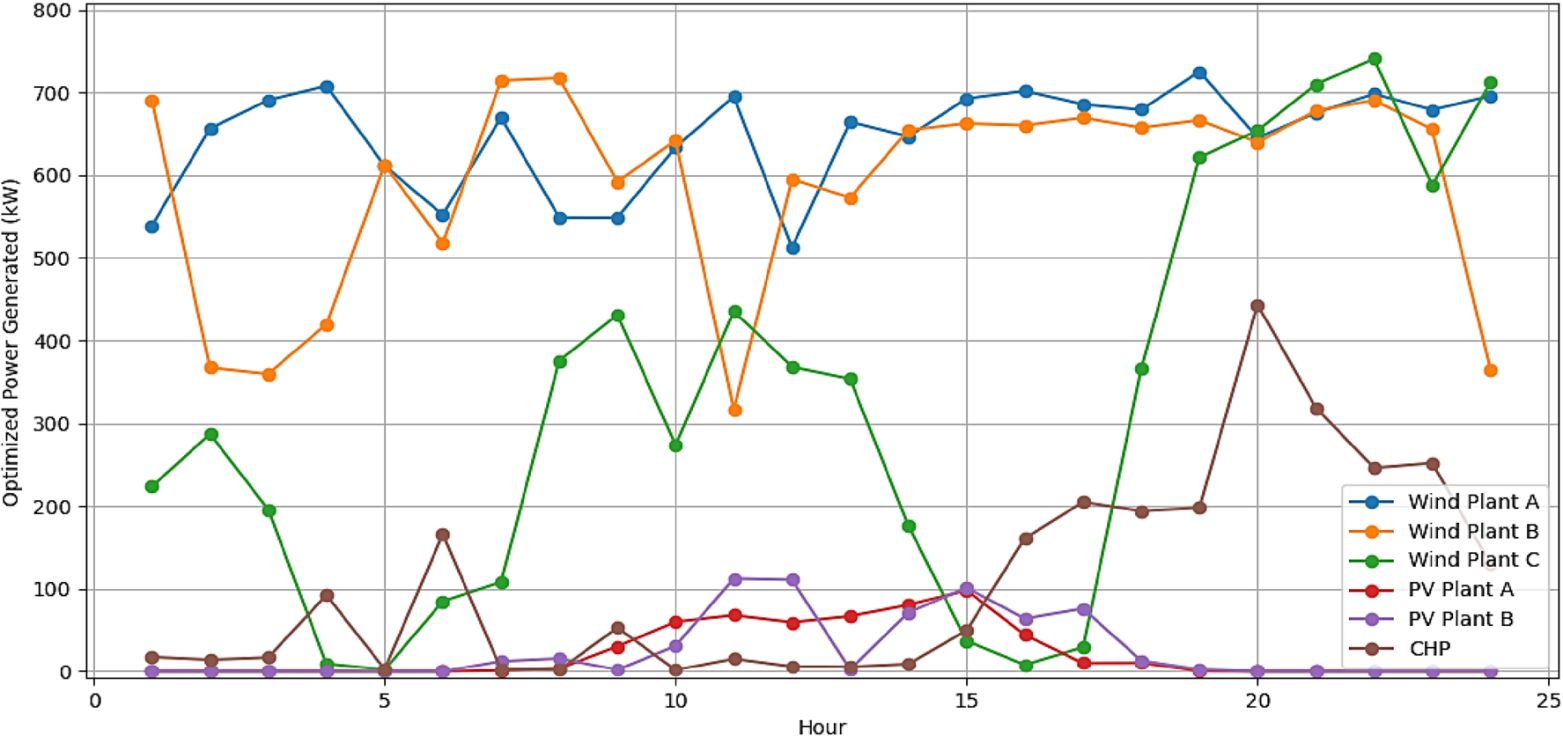
Optimized scheduling using IPOA.

**Figure 10:**
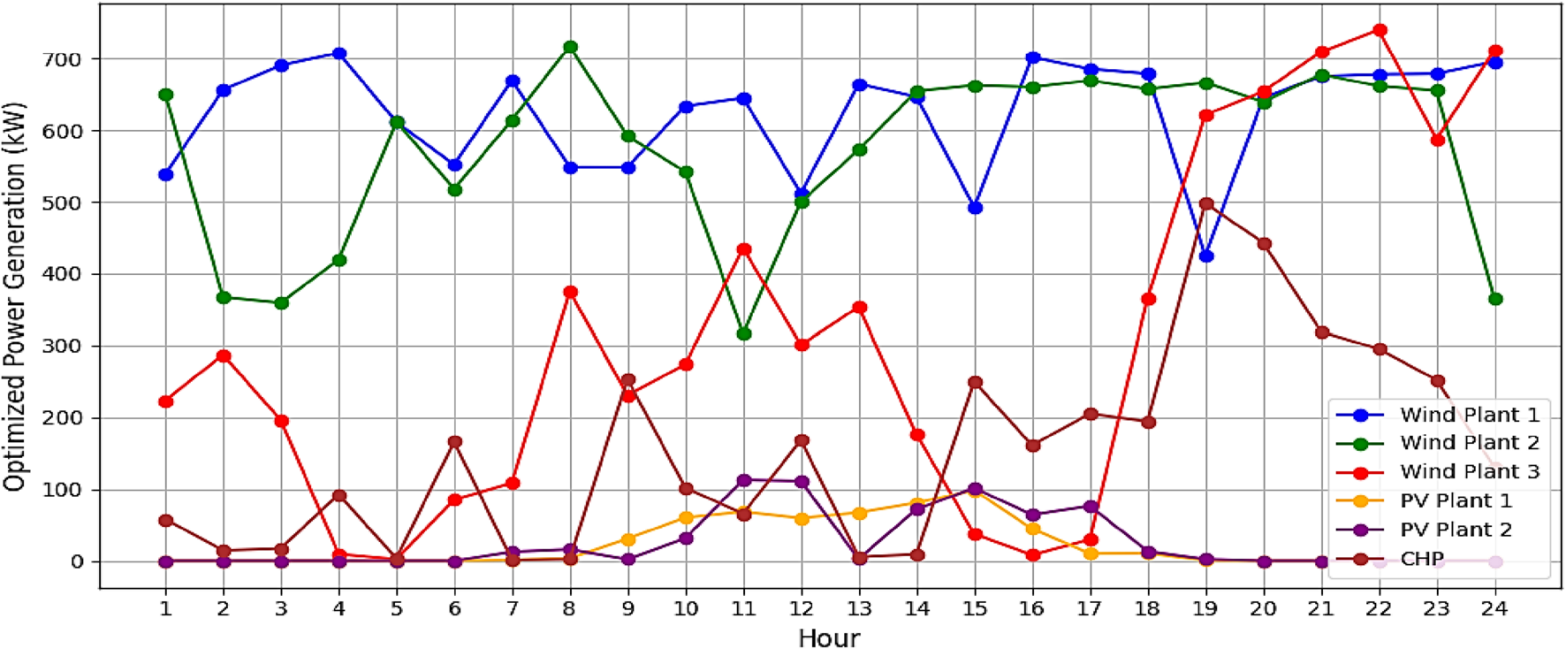
Optimized scheduling using POA.

**Figure 11:**
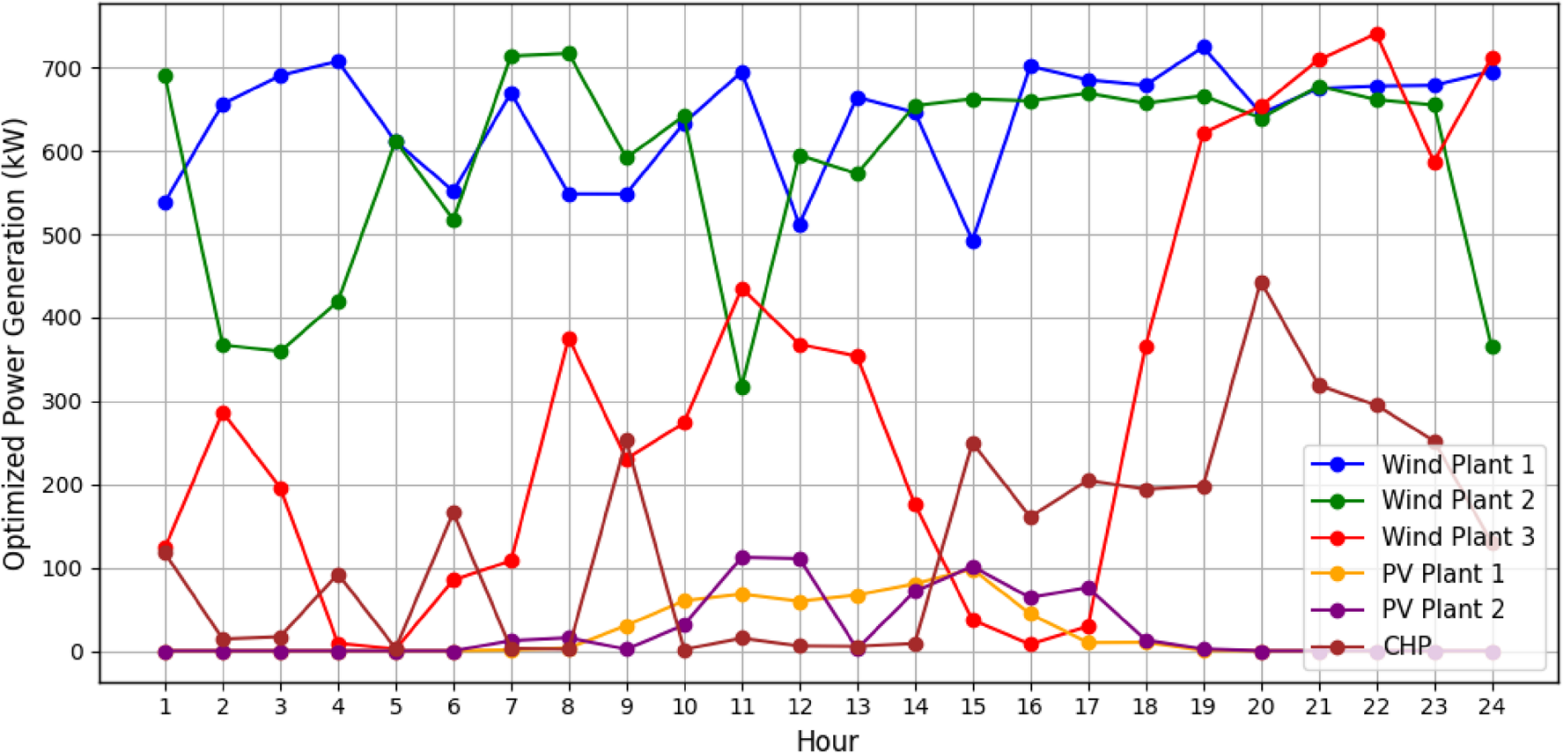
Optimized scheduling using PSO.

**Figure 12:**
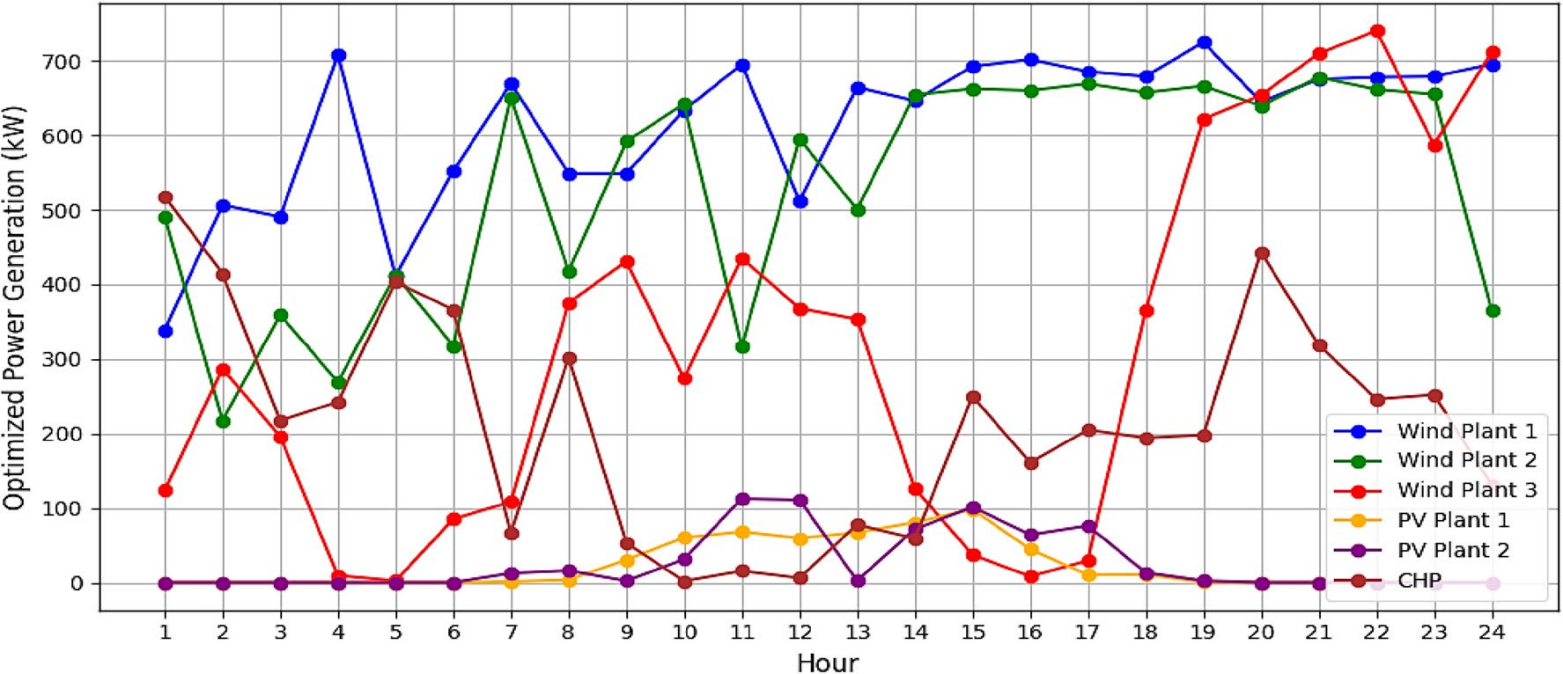
Optimized scheduling using GA.

It is assumed that all the units produce power and are running within permitted bounds. The hourly best scheduling of power generation for all generators utilizing various algorithms are shown in Figs. 9, 10, 11, and 12. The best scheduling utilizing IPOA shown in Fig. 9 reveals a dynamic and efficient allocation of power across the various DERs. The results revealed fluctuations in production across all algorithms, with IPOA demonstrating superior performance in minimizing production costs. Notably, IPOA exhibited reduced reliance on CHP system compared to other algorithms, resulting in lower production cost whiles meeting the grid demand. This highlights the effectiveness of IPOA’s exploration and exploitation strategies in achieving cost efficiency while maintaining adequate generation to meet demand.

The superior performance of IPOA compared to other algorithms can be attributed to its enhanced exploration and exploitation techniques, which enable it to efficiently navigate the search space and identify optimal solutions. This approach directly supports the contributing statement of this paper in section "Literature gap and contributions" that continuous improvements and refinement of optimization algorithms are necessary to achieve optimal results. In this regard, IPOA maintains its energy generation objectives while achieving significant cost reductions by effectively balancing the use of renewable energy sources and reducing dependency on costly operation of the CHP. This highlights the value of improvements harnessed by the IPOA illustrated in Fig. 4, in handling the intricate optimization problem of scheduling the DERs to meet grid demand at the lowest possible cost.

The cost of generation in Table 5 affirms the effectiveness of using the IPOA as compared to other algorithms to satisfy the VPP EM problem in Eq. (4). The algorithm optimally schedules the RERs to meet the grid demand thereby reducing the fuel cost of the CHP and reducing negative environmental impact. This is clear in the minimized generation cost to meet the grid demand.

**Table 5:** Generation cost for case study A.

Algorithm	Ideal solution ($)	Worst solution ($)	Mean ($)
GA	1763.7634	1800	1781.9
PSO	1719.2947	1724.8	1722.05
POA	1606.98	1610.32	1608.65
IPOA (proposed)	1547.949	1600.0023	1573.95

**Figure 13:**
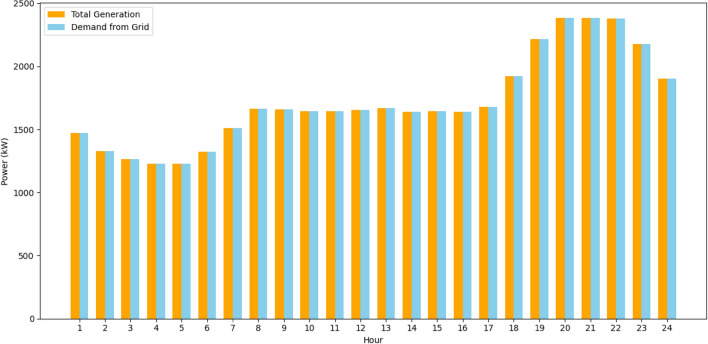
Power balance for Case study A using IPOA.

Figure 13 illustrates the amount of power required by the grid with the amount of power produced by the DERs using IPOA. The IPOA plays a pivotal role by intelligently optimizing the scheduling of DERs within the VPP. Its ability to dynamically balance exploration and exploitation sequences ensures that the VPP adapts in real-time, optimizing the contribution of each DER to keep a harmonized power balance, satisfying Eq. (5) in the VPP EM problem.

B.B. Case study B

**Figure 14:**
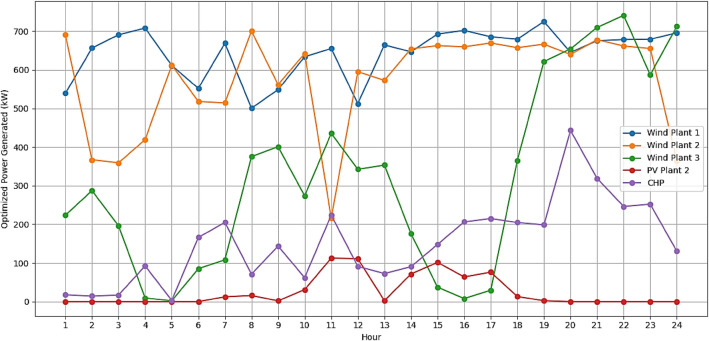
Optimized scheduling using IPOA.

Having established the superiority of IPOA over other algorithms in case A, the power scheduling is studied again to confirm the reliability of the system. Here, reliability is defined as testing the potency of IPOA in optimizing generation from DERs to meet demand in the event of a non-functioning generator. In this regard, the algorithm must dependably and reliably meet demand under this situation, which represents a typical and foreseeable scenario. It is therefore assumed that PV plant A is not generating. The other DERs must provide enough power to match the demand. The hourly best scheduling of power generation applying the IPOA for all generators is shown in Fig. 14. Results, as shown in Table 6, show that there is a spike in the overall generation cost. This is because CHP compensated for the demand deficit, leading to a slight overshoot of the generational cost as compared to case study A. The algorithm proves a keen responsiveness to the unique characteristics of case study B, adjusting the distribution of power among DERs dynamically. This adaptability is a crucial asset in real-world cases where renewable generation and demand patterns may show fluctuations. IPOA’s ability to intelligently navigate these variations reinforces its effectiveness in optimizing the VPP’s operation, ensuring a resilient response to the intricacies of diverse operational conditions and demand dynamics.

**Table 6:** Generation cost Case study B.

Algorithm	Ideal solution ($)	Worst solution ($)	Mean ($)
GA	1785.1722	1805.3564	1795.2643
PSO	1734.5628	1765.8745	1750.2187
POA	1624.78	1704.32	1664.55
IPOA (proposed)	1598.9345	1603.2567	1601.0956

With a mean cost of $1601.0956, IPOA has the lowest cost of all the algorithms when comparing the mean costs of production. POA has a higher mean cost of $1664.55 after IPOA, and the mean costs of PSO and GA are even higher, at $1750.2187 and $1795.2643, respectively.

The cost differential between IPOA and the other algorithms shows how successful IPOA is at maximizing power generation while lowering expenses, even in constrained circumstances such as the offline mode of PV plant A. This implies that IPOA is more capable of adjusting to variations, like the loss of a generator, and effectively distributing available resources to satisfy demand demands, proving its superior reliability and efficiency over other algorithms.

Moreover, the ideal and worst solutions offer additional details on how well each algorithm performs. The algorithm's consistency and dependability in producing optimal outcomes increase with the magnitude of the difference between the ideal and mean solutions. IPOA demonstrates a consistent and resilient power scheduling method with a comparatively low difference between its ideal and mean solutions.

Overall, the findings demonstrate the potential of IPOA as a dependable and practical choice for scheduling power generation, particularly when considering system limitations like generator interruptions. IPOA exhibits its capacity to efficiently manage power generation resources, leading to lower costs and increased system reliability, by utilizing its improved optimization strategies.

C.C. Case study C

**Figure 15:**
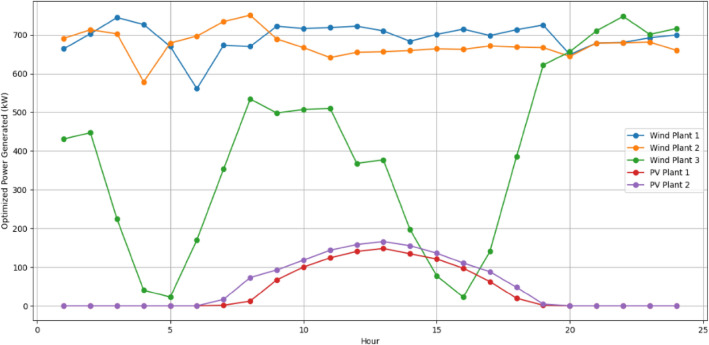
Optimal scheduling for case C using IPOA.

**Figure 16:**
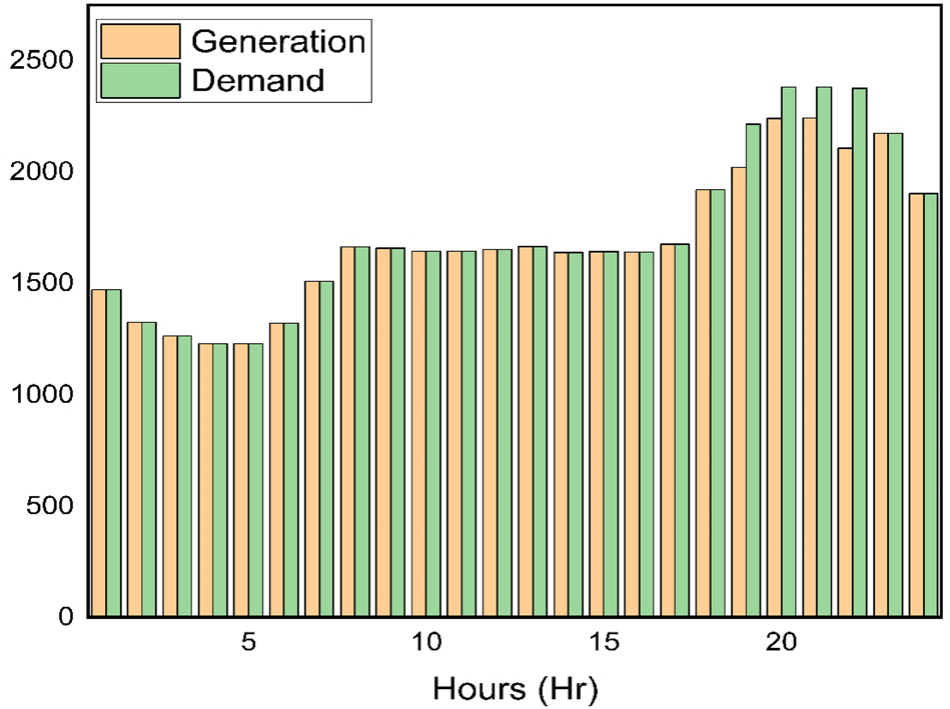
Power balance for case study C.

In this case, it is assumed that the CHP is isolated to again test the reliability of the system. In this case, the idea is to find a scenario where the VPP would not be able to meet demand. Analysis is performed to assess the non-production or the offline state of the CHP unit on the overall output of the VPP. The best scheduling by the algorithm is shown in Fig. 15. The power balance is illustrated in Fig. 16. It could be noted from Fig. 16 that, at some hours of the day, the VPP generation could not meet the demand. Encountering such a scenario underscores robust contingency planning. By diversifying generation sources, the VPP operator can enhance system reliability, minimize disruptions, and ensure uninterrupted delivery to the grid. With this information, the VPP makes informed decisions to govern its operation such as incorporating back-up sources to bridge the gap between demand and supply. This could involve using energy stored in batteries, fuel cells etc. The incorporation of battery energy sources and hydrogen technology to diversify the DER portfolio of the VPP will be studied in the future work of this paper.

The ability of the IPOA to effectively manage DERs within the VPP to fulfil grid demand is what makes it more effective than other algorithms. The IPOA makes sure the VPP operates in a way that balances supply and demand, improving grid stability and dependability, by optimizing generation from DERs with the lowest possible cost. The optimization process continuously shows that IPOA operates well in a variety of settings owing to its improvements in both exploration and exploitation from the basic POA. In Case A, IPOA successfully produced the anticipated outcomes by meeting the demand at the lowest cost compared to other algorithms. Comparably, in Case B, IPOA was still able to meet demand at all hours of the day at the lowest cost of generation, even when PV A was assumed to be offline. Having established the superior performance of IPOA to other algorithms, case C, however, showed a preferred crucial situation, which was the idea in the beginning statement of Case C to find a scenario where the VPP will not be able to meet demand. Obviously, from Fig. 16, the VPP was not able to meet demand at some hours because of the lack of production from the CHP unit. This, however, does not show weakness of IPOA but rather emphasizes the requirement of thorough contingency planning for VPP operators, highlighting the necessity of methods to reduce risks and guarantee system dependability in unanticipated situations.

### Security of optimized data

This section presents the results of securing the optimized generation data from possible cyber threats at the CMS node. The CMS acts as the control mechanism of the VPP which oversees the overall operation of the VPP. Tampering with the data at this point will lead to suboptimal decisions. Through the convolutional autoencoder, the optimized data set is trained for the VPP to learn and recognize normal patterns to help identify abnormal representation.

#### Training of the convolutional autoencoder

**Figure 17:**
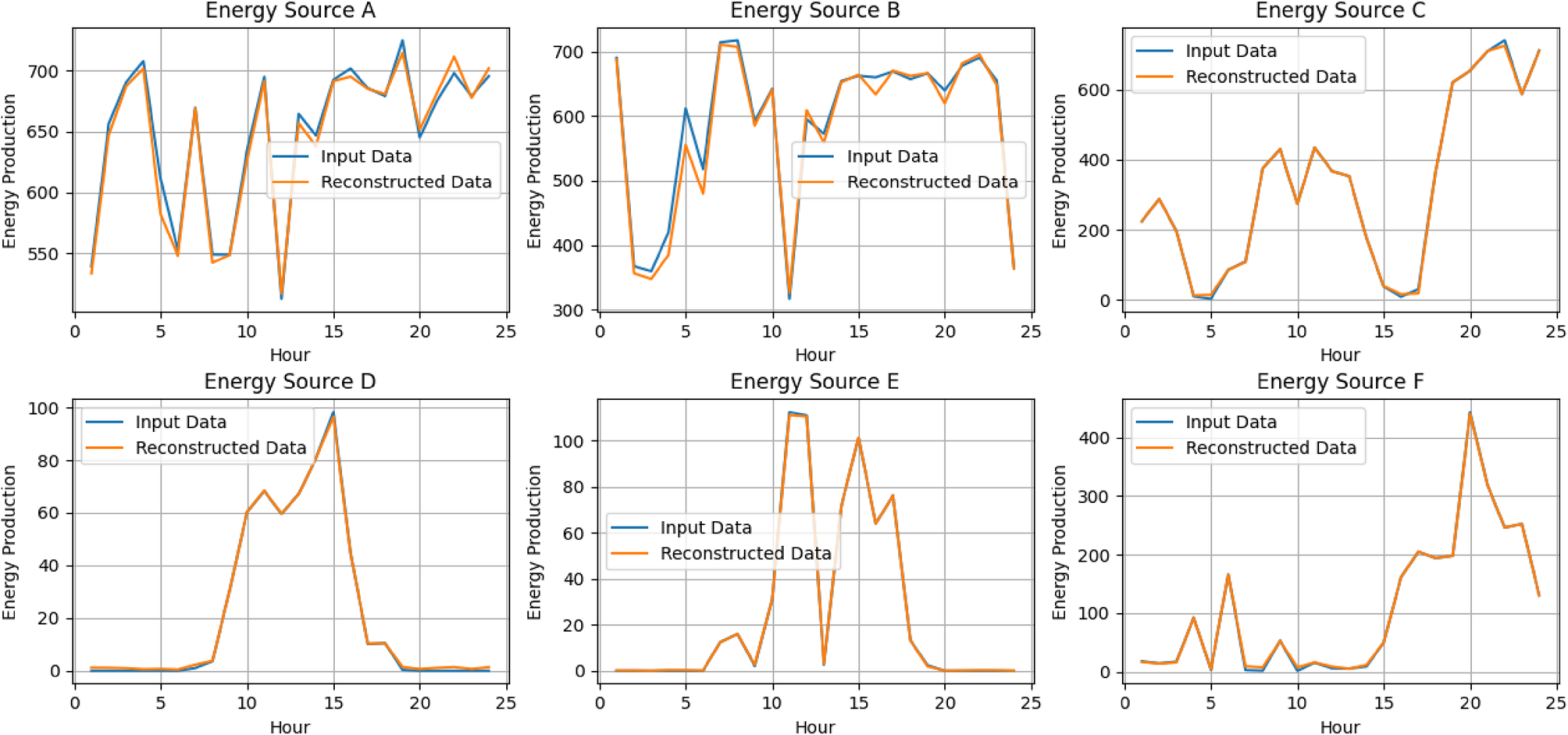
Training of convolutional autoencoder.

The training of the model illustrated in Fig. 17 monitors the system’s behaviour for all the DERs to help detect malicious attempts at manipulating the data from hackers. Training the model to learn from the optimized data is crucial for the CMS to be aware of unseen scenarios and make informed decisions particularly in the face of rising cyber-attacks in modern energy systems like that of the VPP. It is noteworthy to say that, in training the model to learn patterns of the optimized data, there will be losses. In this paper, an 80–20 percentage split for training and validation is adopted. 80% of the data is used to train the model and 20% is set aside for validation. This approach ensures a robust model with a large dataset for learning and a separate set for performance evaluation. This procedure captures diverse patterns and helps identify potential issues, ensuring accurate and trustworthy results.

**Figure 18:**
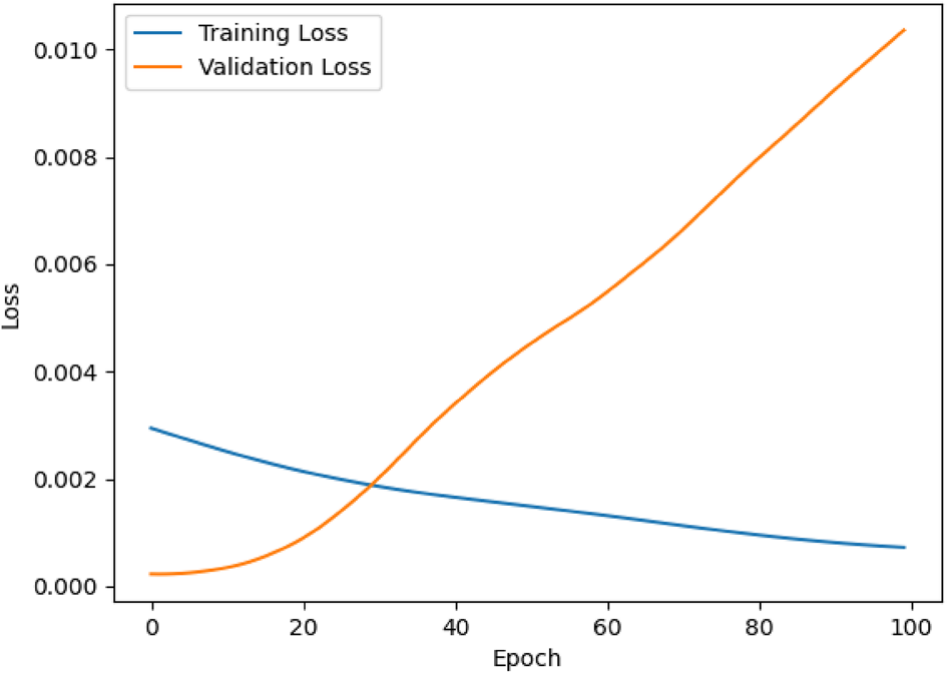
Training and validation loss.

The model's performance during training is evaluated primarily by looking at its training and validation losses. The model's ability to generalize and perform better is shown by lower training and validation losses as illustrated in Fig. 18. Regarding the VPP's defense against cyberattacks, the training and validation loss metrics serve as crucial markers of the data manipulation detection system's resilience and dependability.

#### Data manipulation detection

The outcome shows how well the suggested approach detects data manipulation. Following the completion of the autoencoder's training of the optimized data, the manipulated data, and the reconstructed data by the convolutional autoencoder are compared to find any instances in which the data significantly deviates from the expected values. Applying sensitivity levels of 0.05, 0.10, and 0.15 of deviation of reconstructed data shows varied amounts of manipulation at various times and with different energy sources. The rationale behind providing a sensitivity level in the detection of manipulated data lies in the recognition that not all deviations from normal patterns can be attributed to cyber-attacks. Sensitivity levels help to strike a balance between detecting genuine anomalies, including those caused by cyber-attacks, and minimizing false positives that may arise from fluctuations in energy production since the VPP is continuously monitoring generation from the DERs.

**Figure 19:**
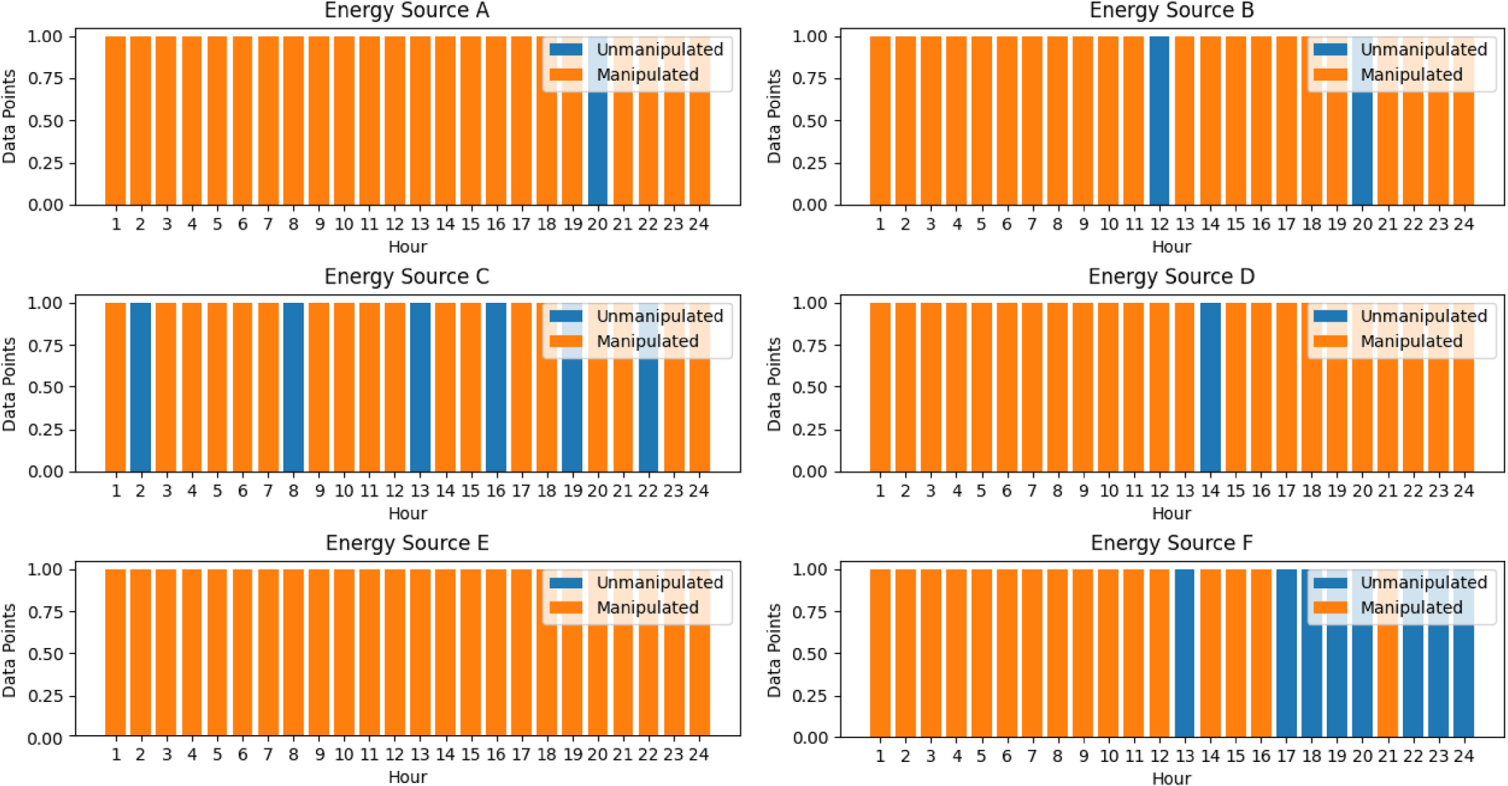
0.05 deviation level.

**Figure 20:**
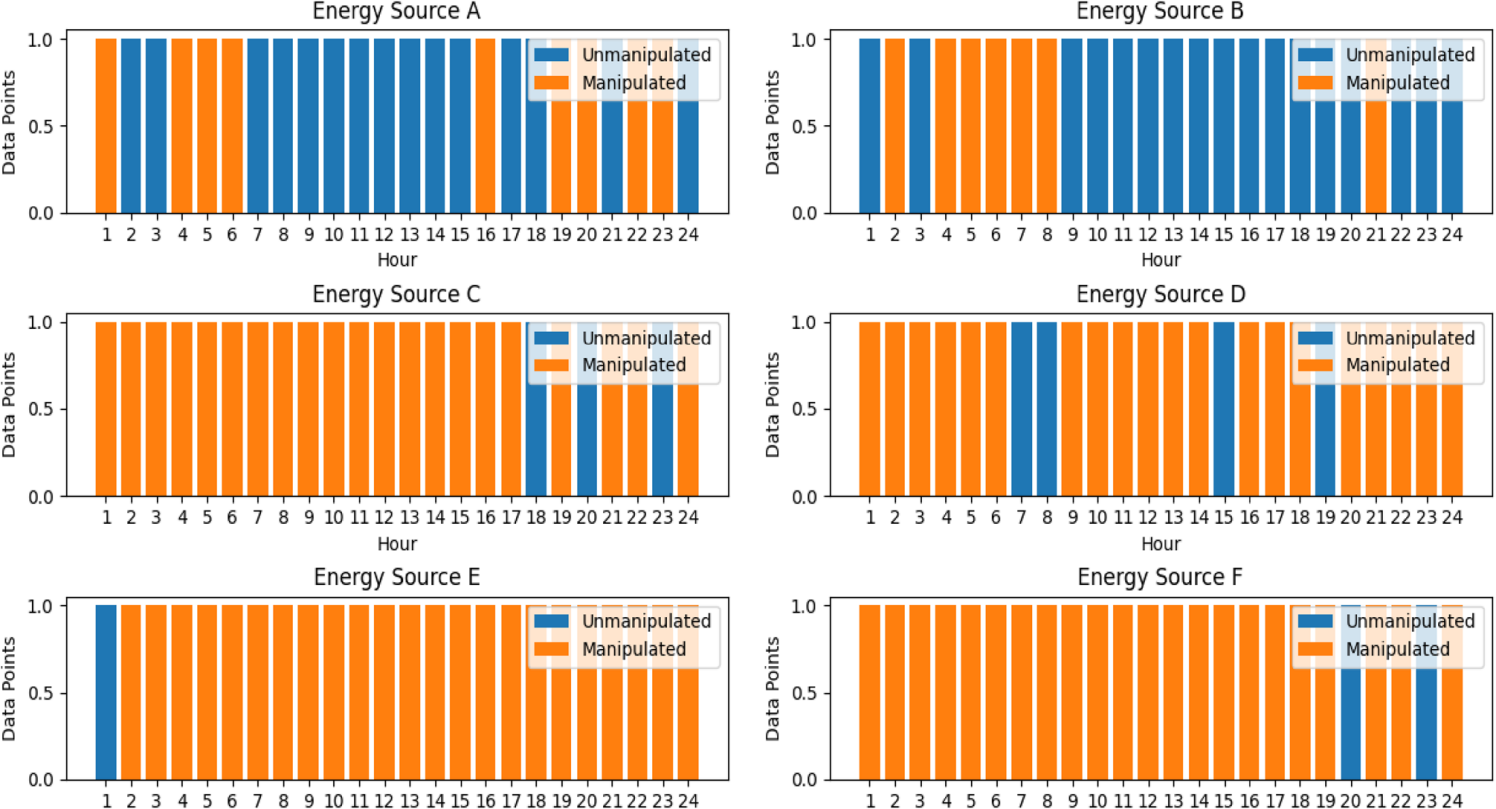
0.1 deviation level.

**Figure 21:**
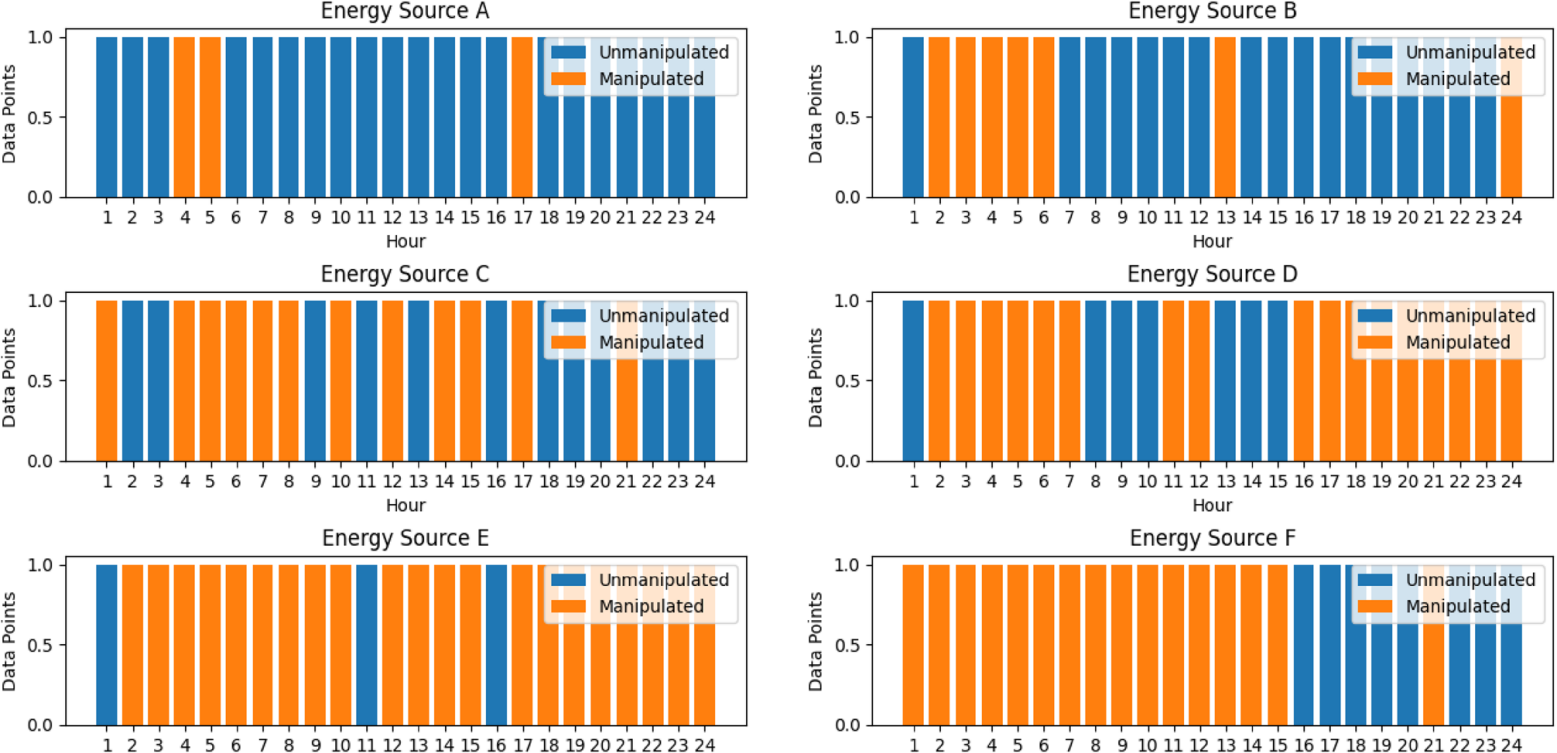
0.15 deviation level.

The manipulation status visualization shown in Figs. 19, 20, and 21 identifies sources and critical times that are vulnerable to manipulation, offering valuable information for risk reduction and data integrity monitoring.

#### Evaluation of convolutional autoencoder

The convolutional autoencoder-based model for detecting manipulated data is evaluated against established detection techniques as shown in Table 7 using two key metrics: False Positive Rate (FPR) and Detection Accuracy (DA). The FPR measures the proportion of negative cases incorrectly classified as positive compared to the total number of reported negative cases. In contrast, the DA is the percentage of accurately identified cases across all classifications, encompassing both manipulated and unmanipulated instances. These metrics offer insights into the model's performance across various identification categories, providing valuable information on its overall effectiveness. In comparison with other methods like Support Vector Machine (SVM), k-Nearest Neighbor (k-NN), and Triangular-based Nearest Neighbor (TANN), the autoencoder model proves superior performance. The table indicates that convolutional autoencoder model achieves higher DA and FPR compared to these methods^54^.

**Table 7:** Comparison between different manipulated data detection models.

Detection method	FPR (%)	DA (%)
k-NN^54^	38.02	88.91
SVM^54^	6.91	92.98
TANN^54^	3.83	96.91
Autoencoder (generic)	2.09	97.89
Convolutional autoencoder (proposed)	1.92	98.06

### Economic analysis

For a thorough cost–benefit analysis of the system, all expenses and revenue associated with operating the VPP must be thoroughly assessed. The evaluation of the system's viability and potential returns on investment is aided by the analysis. The cost–benefit analysis in this paper focuses on the revenue generated from supplying the power demand to the grid and the cost of generating the power. This is done by examining the impact of price volatility, which gauges how much prices fluctuate over time.

#### Day-ahead market

The DAM is a crucial component of electricity markets, providing a platform for the VPP to trade its energy production with the grid for the following day. As a measure to mitigate financial shocks described in Eq. (24), the VPP makes a DAM price forecast using the Prophet tool which serves as a signal about the expected market conditions. These signals influence decision-making, investment strategies, and the overall behavior of market conditions. The major focus of the VPP when making pricing decisions is to balance their profit with risks since there are many uncertainties when making pricing decisions. The risks come from the uncertainty brought by the RES generation deviation and the volatility of the spot prices.

#### Price volatility mitigation using prophet

**Figure 22:**
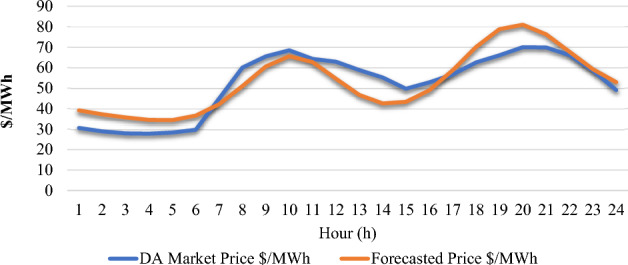
Prophet price forecast.

**Figure 23:**
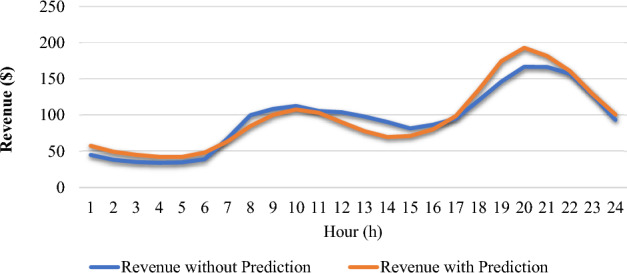
Hourly revenue from price forecast.

**Figure 24:**
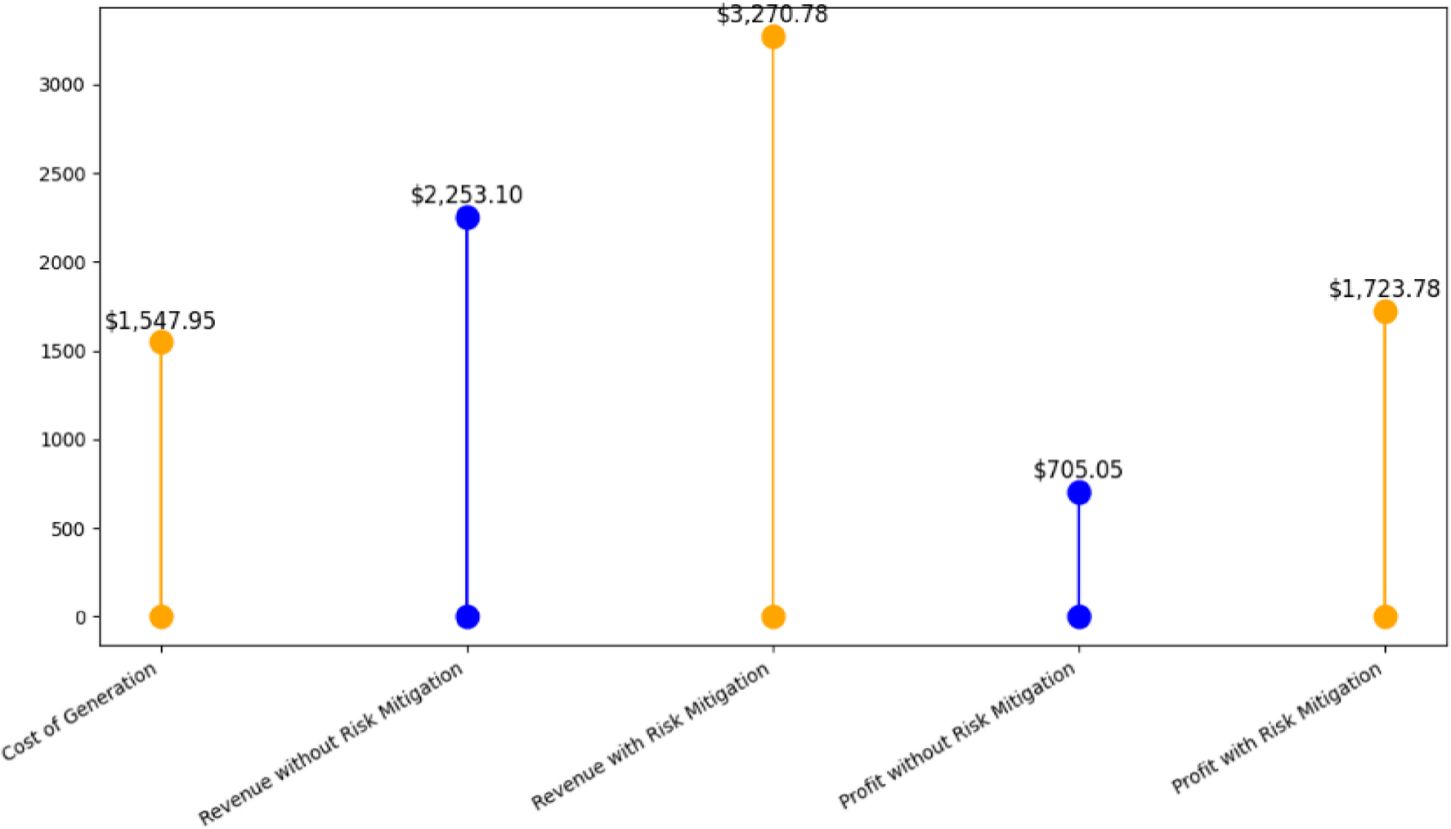
VPP economic performance.

The Prophet model becomes invaluable in managing price volatility in the energy market by accurately forecasting short- to medium term trends using past data, offering timely insights for informed decision-making. In contrast to scenario-based models, it offers a detailed and data-driven method that is adapted to the dynamic nature of the DAM, allowing for quick adjustments in reaction to changing circumstances as illustrated in Figs. 22 and 23, respectively. Consequently, economic performance considering revenue generated with and without the use of the price volatility mitigation approach is shown in Fig. 24.

In summary, the economic performance in terms of cost generation, revenue gained with and without price prediction underscores the critical importance of utilizing the price volatility tool (Prophet) in the DAM. The model aims to predict price trends, enabling more informed decision-making in market participation. The substantial increase in revenue and profit with price forecast highlights the significant financial risk mitigation achieved using the model, emphasizing its role in optimizing the VPP’s economic gains within the context of DAM.

## Conclusions

This paper proposed an investigation for an efficient operation of VPP. The investigation comprises of a tri-level approach. The first approach is for the VPP to optimally schedule its DERs to meet grid demand by employing an improved pelican optimization algorithm. The continuous improvement of optimization algorithms underscores the necessity of refinement for enhanced performance leading to better solutions and more accurate results. To arrive at the improved pelican optimization algorithm, a detailed analysis of the fundamental pelican optimization algorithm’s underlying architecture is conducted. Then, to more closely mimic the hunting behaviour seen in pelicans, three different motion techniques are presented, replacing the earlier motion approach of the generic pelican optimization algorithm. Through the combination of these three unique motion techniques, the IPOA presents a novel strategy for resource allocation optimization in VPP. The benefits of IPOA can be attributed to its capacity to address the limitations of the standard POA and produce superior optimization outcomes, as demonstrated by the results. In this regard, the algorithm showed enhanced effectiveness compared to other well established optimization algorithms by meeting the required demand at a lower production cost of 8.5%. In the second stage, the VPP adopted an approach to secure the optimized data from the optimization process as a measure to mitigate the adverse effects of cyber threats. While existing literature often overlooks cybersecurity concerns within VPPs, focusing only on the market and resource management, it was imperative to address this gap. Recognizing the vulnerability of VPPs to cyber threats, this approach employed a machine learning algorithm, preferably, the convolutional autoencoder to learn patterns of the optimized generational data with the aim of flagging or detecting manipulated data from the data streams at the central management system of the VPP. Also, the convolutional autoencoder showed improved performance against the traditional autoencoder and other well-known detection techniques with a detection accuracy and false positive rate of 98.06% and 1.92% respectively. Finally, in the last level of the operation, the VPP engaged the energy market to sell its optimized generated power to the grid. In this stage, the importance of assessing the risk of price volatility in the energy market where the VPP operates is studied. Notably, existing research predominantly focuses on profit maximization and increased bids in the energy market without adequately addressing the associated risks. To mitigate the risk of price volatility for a more realistic profit maximization, the VPP employed the Prophet algorithm to mitigate the negative effects of price volatility in the energy market. Analysis of economic performance showed the usefulness of the Prophet algorithm to aid the VPP in maximizing its revenue and profits in the dynamic energy market, thereby providing resilient economic viability, and paving the way for scalability.

This tri-level coordinated approach addresses key challenges in the energy sector, including resource optimization, cybersecurity, and market volatility, thereby facilitating progress towards achieving universal access to clean and affordable energy.

Future research could explore several promising directions to advance the field of Virtual Power Plants and energy system optimization, building upon the findings and methodologies presented in this manuscript. First, to improve the effectiveness and scalability of VPP operations, more research is required into the integration of proficient optimization algorithms, such as hybrid approaches, genetic algorithms, and particle swarm optimization. These algorithms could be modified to handle problems like changing market dynamics, grid constraints, and the increasing adoption of renewable energy sources. Second, the implementation of machine learning and artificial intelligence approaches need additional research, particularly in the context of predictive maintenance, fault detection, and anomaly identification in VPP infrastructure. Using advanced data analytics and predictive modelling methodologies could enable proactive maintenance plans that improve asset performance and longevity while reducing downtime and operational disturbances. Furthermore, future research could focus on developing comprehensive cybersecurity frameworks specifically tailored to VPPs, encompassing intrusion detection, secure communication protocols, and resilience against cyber threats. As VPPs become more interconnected and data-driven, ensuring the integrity, confidentiality, and availability of critical infrastructure and information systems is paramount to safeguarding against potential cyber-attacks and malicious activities.

## Data Availability

The datasets used and/or analysed during the current study available from the corresponding author on reasonable request.

## Abbreviations

ACO: Ant colony optimization
AV: Attacking vector
BOA: Butterfly optimization algorithm
CDEA: Compound differential evolution algorithm
CHP: Combined heat and power
CMS: Central management system
CRSTS: Central receiver solar thermal system
CVaR: Conditional value at risk
DA: Detection accuracy
DAM: Day ahead market
DER: Distributed energy resource
EM: Energy management
EV: Electric vehicle
FPR: False positive rate
GA: Genetic algorithm
GD: Genuine data
HHO: Harris Hawk optimization
IPOA: Improved Pelican optimization algorithm
K-NN: K- Nearest neighbour
LB: Lower bounds
MD: Manipulated data
PDF: Probability density function
POA: Pelican optimization algorithm
PSO: Particle swarm optimization
RD: Ramp down
RER: Renewable energy resource
RU: Ramp up
SA: Simulated annealing
SCADA: Supervisory control and data acquisition
SDG: Sustainable development goal
SVM: Support vector machine
TANN: Triangular based nearest neighbour
UB: Upper bounds
VaR: Value at risk
VPP: Virtual power plant
WT: Wind turbine

## List of symbols

η: Efficiency

## ${P}_{j}^{max}:\text{ Maximum power generation of each DER}$

$${P}_{LS}$$: Power loss
$${P}_{L}$$: Load demand
${P}_{rCHP}$: Rated power of CHP
${P}_{WT}$: Power output of wind Turbine
${B}_{kj}$: Coefficient of bus k and j
${B}_{KO}$: Loss coefficient of buses
${B}_{oo}$: Fixed transmission loss term
${V}_{L}$: Cut-in speed of wind Turbine
${V}_{H}$: Cut-out speed of wind Turbine
$${P}_{PV}$$: PV power
$$(\overrightarrow{{X}_{j}^{k}})$$: Pelican position
$\overrightarrow{{X}_{j,d}^{t+1}}$: Improved Pelican position
${P}_{Panel}$: Maximum output of each PV Panel
$${I}_{s}$$: Solar Irradiance
